# High-throughput virome profiling reveals complex viral diversity, co-infection patterns, and novel viruses in *Cnidium officinale* in Korea

**DOI:** 10.3389/fpls.2025.1644750

**Published:** 2025-12-10

**Authors:** Jeong-Hun Kang, Aamir Lal, Minhyeok Kwon, Myeonghwan Kwak, Chung Ryul Jung, Eui-Joon Kil

**Affiliations:** 1 Department of Plant Medicals, Gyeongkuk National University, Andong, Republic of Korea; 2 Agriculture Research Institute, Gyeongkuk National University, Andong, Republic of Korea; 3 Special Forest Resources Div, Forest Bioresources Dept, National Institute of Forest Science (NIFoS), Suwon, Republic of Korea

**Keywords:** *Cnidium officinale*, virome, high-throughput sequencing (HTS), novel viruses, apple stem grooving virus (ASGV), cucumber mosaic virus (CMV)

## Abstract

High-throughput sequencing (HTS) was used to comprehensively profile the virome of *Cnidium officinale* Makino, an economically and medicinally important herb widely cultivated in Korea. Symptomatic leaf samples from nine major cultivation regions were pooled for sequencing. The analysis detected a diverse assemblage of viruses comprising 31 distinct species, including apple stem grooving virus (ASGV), cucumber mosaic virus (CMV), cnidium vein yellowing virus-1 (CnVYV-1), and cnidium virus X (CnVX). Importantly, four novel viral agents were identified: Cnidium officinale virus 1 (CnoV1), cnidium virus Y (CnVY), cnidium virus Z (CnVZ), and a unique cnidium virus–associated satellite RNA (satCnV). Viral read distributions varied markedly by regions; for example, CMV dominated in Bonghwa (two sites) and Geochang, while CnVY prevailed in Samcheok-B and Jecheon. Phylogenetic analyses of ASGV and CMV isolates from *C. officinale* revealed distinct lineages unique to this host, suggesting host-specific adaptation. Co-infection patterns indicated notable interspecific viral interactions. In particular, we observed a strong antagonism between CnVY and CMV, suggesting competitive exclusion or mutual suppression. A virus co-occurrence network analysis identified additional significant virus–virus associations, and principal component analysis (PCA) of virome profiles showed that samples cluster according to their dominant viruses. Overall, our findings emphasize the power of HTS in uncovering complex viromes and advancing understanding of virus ecology in clonally propagated medicinal crops. The comprehensive virome dataset generated here provides an essential foundation for future epidemiological studies and management strategies in *C. officinale* and related species.

## Introduction

*Cnidium officinale* Makino (family Umbelliferae), known as “Cheongung” in Korea, “Senkyu” in Japan, and “Chuanxiong” in China, is an economically important and culturally significant medicinal herb widely cultivated in East Asia (Kim et al., 2020). Its rhizomes exhibit anti-inflammatory, analgesic, and antioxidant activities, which have made *C. officinale* a staple in traditional medicine (Bae et al., 2011). These therapeutic effects are attributed to high levels of bioactive compounds such as phthalides, coumarins, and essential oils (Oh et al., 2010). Because *C. officinale* is propagated vegetatively, viruses can accumulate with each planting cycle, threatening both rhizome yield and the levels of active compounds (Bae et al., 2011). To date, virus-free propagation programs (e.g., clean seed-rhizome production) are lacking, in part because the full spectrum of viruses infecting this crop has not yet been fully characterized.

Despite its importance, the virome of *C. officinale* remains poorly characterized, with only a few viruses reported to date. For example, studies in Korea identified cnidium vein yellowing virus-1 (CnVYV-1) and -2 (CnVYV-2) associated with vein-yellowing symptoms (Yoo et al., 2015). A recent HTS-based analysis significantly expanded our knowledge by identifying additional viruses, but it focused on a single region and lacked detailed characterization of genome structures and regional distribution (Belete et al., 2024). Comprehensive virome characterization using HTS can provide critical insights into the diversity, evolutionary dynamics, and epidemiology of viruses infecting medicinal crops (Roossinck et al., 2015; Massart et al., 2017). Accurate identification of virus diversity and distribution is crucial for developing effective management strategies. This is especially true for medicinal crops like *C. officinale*, whose pharmacological quality can be directly impacted by viral infections – in particular, the timing of infection (Jeong et al., 2014; Fraile and García-Arenal, 2016; Mehetre et al., 2021).

Complex virus co-infections in plants can significantly alter host–virus interactions, symptom expression, transmission dynamics, and overall pathogenicity, influencing crop productivity and quality (Hadidi et al., 2016). HTS enables comprehensive, unbiased detection of both known and novel viruses without prior genomic information, making it particularly suitable for virome studies (Hadidi et al., 2016, 2012; Barba et al., 2014; Roossinck et al., 2015; Wu et al., 2015; Maree et al., 2018; Villamor et al., 2019; Maclot et al., 2020; Kutnjak et al., 2021; Maina et al., 2024). Modern plant viromics has revealed extensive viral diversity across hosts and environments and has accelerated discovery and diagnostics (Roossinck et al., 2015; Minicka et al., 2020). In practice, HTS can be implemented using rRNA-depleted total RNA, dsRNA or VANA enrichments, small-RNA sequencing, or, where appropriate, long−read platforms for rapid or field-deployable analyses; target−enrichment (hybrid-capture) can further improve sensitivity for low−titer viruses (Pooggin, 2018; Boykin et al., 2019; Sun et al., 2022; Briese et al., 2015; Farrall et al., 2024). Adoption in plant-health programs is supported by international guidelines that address validation, controls, and reporting (Massart et al., 2022).

In this study, we utilized HTS to systematically investigate the virome of symptomatic *C. officinale* plants from nine distinct geographic regions of Korea. Our specific objectives were: (i) to catalog the known and novel viruses infecting *C. officinale*; (ii) to quantitatively assess the relative abundance (RPKM) and distribution of identified viruses across different cultivation regions; and (iii) to elucidate the evolutionary relationships, genetic diversity, and complex co-infection patterns (e.g., antagonism and co-occurrence) of predominant viruses – particularly ASGV and CMV – through detailed phylogenetic and population genetic analyses. These findings provide valuable baseline data for further research into virus epidemiology and host–virus interactions. Moreover, they inform effective disease management strategies, ultimately enhancing the sustainable production and medicinal quality of *C. officinale*.

## Materials and methods

### Sample collection

Symptomatic leaves showing vein-yellowing, chlorosis, or mosaic were collected from nine cultivation sites across five provinces in Korea. These included Geundeok-myeon, Samcheok (SC-A) and Hajang-myeong, Samcheok (SC-B) in Gangwon State; Hoengseong (HS) and Pyeongchang (PC), also in Gangwon State; Myeongho-myeon, Bonghwa (BH-A) and Socheon-myeon, Bonghwa (BH-B) in Gyeongsangbuk-do; Geochang (GC) in Gyeongsangnam-do; Jecheon (JC) in Chungcheongbuk-do; and Jinan (JA) in Jeonbuk State (**Supplementary Figure S1**; **Supplementary Table S1**). For each region, we generated one pooled library from at least five symptomatic plants. From each plant, at least three leaves were collected to capture within-site virus diversity. Due to this pooling strategy, subsequent analyses focused on regional virome community structure and not on single-plant virus–symptom correlation. Representative symptoms observed in the field – including vein-yellowing in Samcheok-B and chlorosis in Jecheon – are illustrated in **Supplementary Figure S1**, together with a map showing the nine sampling sites across Korea. All collected tissues were immediately frozen in liquid nitrogen and stored at –80 °C until processing.

### RNA extraction, library preparation, and high-throughput sequencing

Total RNA was extracted from approximately 100 mg of pooled symptomatic leaf tissue per site using the RNeasy Plant Mini Kit (Qiagen, Hilden, Germany) according to the manufacturer’s instructions. RNA integrity and concentration were assessed with a Bioanalyzer 2100 (Agilent Technologies, Santa Clara, CA, USA). For each of the nine sites, an RNA sequencing library was constructed using the TruSeq Stranded Total RNA Sample Prep Kit (Illumina, San Diego, CA, USA), including ribosomal RNA depletion to enrich for messenger and viral RNAs. The libraries were indexed, pooled in equimolar amounts, and sequenced on an Illumina NovaSeq 6000 platform, generating 150 bp paired-end reads. All raw sequencing data have been deposited in the NCBI Sequence Read Archive under BioProject accession PRJNA1031353.

### Transcriptome assembly and virus identification

Adapter trimming and quality filtering were performed on raw reads using Trimmomatic (Bolger et al., 2014). The high-quality reads from each library were then *de novo* assembled using CLC Genomics Workbench v23.0.4 (Qiagen, Hilden, Germany) (Haas et al., 2013). Assembled contigs were screened by tBLASTx against the NCBI RefSeq viral genome database. Putative viral contigs were further validated against an in-house plant virus database to refine virus identification. For sequences showing high similarity to known viruses, we also performed BLASTn (E-value ≤ 1×10^−5^) against a curated plant virus sequence database (Jo et al., 2018). The absolute read counts per virus in each library are summarized in **Table 1**. To account for differences in viral genome length and sequencing depth, we normalized read counts to RPKM (reads per kilobase per million mapped virus reads) using the equation given in **Table 2**. These RPKM values served as input for all downstream relative abundance and diversity analyses. HTS read counts (or RPKM) are semi-quantitative and reflect relative abundance; they should not be equated directly with biological “titer.” Normalization and thresholds are described in the Bioinformatics section, and reporting follows recent HTS diagnostic guidelines (Massart et al., 2022). Applying standard ICTV species demarcation criteria (Lefkowitz et al., 2018), we identified four contigs as representing novel viruses. The complete genomes of these putative new viruses have been deposited in GenBank (accession numbers provided in **Supplementary Table S2**).

**Table 1:** Raw read counts of all viruses identified across nine *Cnidium officinale* libraries.

Name	BH-A	BH-B	SC-A	SC-B	HS	GC	JC	PC	JA
Angelica bushy stunt virus	439	260	835	386,349	154	67	13,958	90,407	317
Apple stem grooving virus	6,871	132,433	106,008	–	25,163	13,344	25,737	55,059	147,038
Bean golden yellow mosaic virus	8	10	24	43	4	–	1	–	7
Beet curly top virus	46	33	32	35	19	5	–	4	18
Carrot red leaf virus	–	–	–	–	3	–	–	–	7
Cauliflower mosaic virus	21	4	2	24	1	–	2	48	51
Cnidium officinale virus 1	13,192	2,565	41,253	7,043	2,230	15,205	6,678	27,531	9,630
Cnidium polerovirus 1	4,009,136	46,306	4,415,497	105,301	5,142,227	295,080	207,406	11,515	106,011
Cnidium vein yellowing virus-1	245,358	476,886	2,394,665	251,763	74,934	742,277	46,319	159,615	153,732
Cnidium vein yellowing virus-2	417,461	483,229	2,364,933	188,014	61,905	405,488	34,699	72,000	125,683
Cnidium virus 1	3,173	102,299	323,121	196,486	6,000,309	240,269	346,597	54,279	590,578
Cnidium virus 2	36,844	11,888	98,345	34,354	280,763	34,319	57,639	21,228	75,086
Cnidium virus X	584,314	233,075	867,608	78,618	6,027,021	1,036,680	119,196	330,130	549,189
Cnidium virus Y	2,330,165	1,014,608	3,268,677	18,355,578	35,725,784	1,540,714	12,201,629	21,425,130	6,221,915
Cnidium virus Z	263,962	78,770	610,635	336,354	380,844	276,328	269,231	93,201	132,364
Cnidium virus-associated satellite RNA	506,727	693,582	4,580,052	285,199	69,877	1,418,706	33,874	210,900	208,965
Cnidoscolus mosaic leaf deformation virus	48	2	70	58	14	5	–	1	9
Cucumber mosaic virus	38,052,052	71,816,719	28,576,487	324	11,776,344	31,099,505	560,408	16,922,486	16,325,560
Iris domestica betaflexivirus 1	11,525	1,066	38,363	6,163	18,216	1,640	1,470	2,020	6,176
Jujube mosaic-associated virus	23	3	18	67	44	–	7	2	24
Lettuce necrotic leaf curl virus	383	43	6,292	863	2,195	115	159	197	737
Lychnis mottle virus	19,695	62,702	206,449	33,814	12,353	57,828	3,456	14,438	12,108
Raspberry latent virus	–	–	–	–	–	–	–	–	5
Strawberry polerovirus 1	37	3	25	20	19	–	1	–	8
Strawberry virus 2	–	450	8	191	90	–	6	2	297
Sweet potato leaf curl virus	11	9	–	10	11	–	–	1	7
Taro bacilliform CH virus	37	25	54	16	18	–	17	3	15
Turnip leaf roll virus	49	10	29	31	26	16	16	–	6
Turnip yellows virus	–	–	–	–	–	2	–	–	1
Wild carrot red leaf virus	4	–	–	–	3	–	–	–	–

**Table 2:** RPKM values* for the top 15 most abundant viruses infecting *Cnidium officinale*.

Name	BH-A	BH-B	SC-A	SC-B	HS	GC	JC	PC	JA
AnBSV	1.14	0.42	2.1	2296.93	0.28	0.22	120.74	275.83	1.55
ASGV	22.75	271.3	340.79	0	59.06	55.26	284.5	214.67	917.87
CnoV1**	31.18	3.75	94.65	38.19	3.74	44.94	52.69	76.61	42.91
CnPV1	14157.03	101.17	15138.78	853.22	12871.9	1303.3	2445.15	47.88	705.77
CnVYV-1	507.05	609.77	4804.96	1193.86	109.78	1918.68	319.58	388.42	598.98
CnVYV-2	860.08	615.98	4730.75	888.83	90.41	1044.92	238.67	174.67	488.19
CnV-1	4.87	97.21	481.84	692.45	6532.69	461.56	1777.2	98.16	1710.08
CnV-2	58.57	11.69	151.8	125.32	316.41	68.24	305.93	39.74	225.05
CnVX	2106.92	519.99	3037.49	650.47	15405.42	4675.49	1434.91	1401.72	3733.49
CnVY**	4549.26	1225.6	6196.07	82229.8	49443.12	3762.34	79530.79	49255.25	22901.81
CnYZ**	583.7	107.77	1311.05	1706.68	596.99	764.29	1987.64	242.69	551.84
satCnV**	11029.49	9340.6	96792.58	14244.16	1078.17	38623.88	2461.57	5405.47	8575.25
CMV	94898.25	110815.7	69195.69	1.85	20819.04	97009.77	4666.03	49695.76	76760.89
IrBFV1	49.08	2.81	158.62	60.22	54.99	8.74	20.9	10.13	49.58
CnVYV	38.45	75.74	391.34	151.48	17.1	141.21	22.53	33.19	44.57

*The RPKM (Reads Per Kilobase per Million mapped reads) values were calculated to quantify relative virus abundance normalized by genome length and total reads. Higher RPKM values indicate higher relative abundance across the libraries ( $\text{RPKM}=\frac{\text{reads }\times 10^{6}}{\text{library}-\text{level virus}-\text{mapped reads}[\text{milion}]\times \text{genome length}[\text{kb}]***}$).

**Novel viruses identified in *C. officinale* in this study.

RPKM values were calculated based on genome lengths obtained from complete genome sequences available in the NCBI GenBank database. For novel viruses identified in this study (marked with “**”), genome lengths were estimated based on the closest known related viruses with complete genome sequences.

### Validation of HTS data by RT-PCR and sanger sequencing

To validate the HTS findings, virus-specific primers were designed from the assembled viral contigs (see **Supplementary Table S3** for primer sequences). Complementary DNA was synthesized and PCR amplification was performed using the SuPrimeScript RT-PCR Premix (Genetbio, Daejeon, Korea) according to the manufacturer’s instructions. The RT-PCR products (300–800 bp) were analyzed by agarose gel electrophoresis (**Supplementary Table S4**), gel-purified, and bidirectionally Sanger sequenced (Macrogen, Seoul, Korea). All amplicon sequences matched the HTS-derived contigs with ≥ 99% identity, confirming the presence of each HTS-identified virus in the original samples.

### Phylogenetic and sequence diversity analysis

Phylogenetic trees were constructed from complete or near-complete viral genome sequences to assess evolutionary relationships. Reference sequences from diverse hosts and regions were retrieved from GenBank and aligned with the *C. officinale* virus sequences using MUSCLE and/or ClustalW. Neighbor-joining trees (1,000 bootstrap replicates) were inferred in MEGA11 (Nei and Kumar, 2000; Kumar et al., 2016) to visualize how *C. officinale* virus isolates cluster relative to known strains. We also computed pairwise nucleotide identities using the Sequence Demarcation Tool v1.3 (SDT) for key viruses, and performed sliding-window analyses of nucleotide diversity (π) for selected viruses (ASGV and CMV) using DnaSP v6.12.03 (Librado and Rozas, 2009; Rozas et al., 2017).

### Statistical analysis and virus co-occurrence network

All statistical analyses were conducted in R v4.1.0. We first calculated Spearman’s rank correlation coefficients to assess pairwise associations between viruses’ relative abundances (RPKM values) across the nine libraries. The vegan package in R (Oksanen et al., 2019) was used to compute the correlation matrix. Only strong correlations (|ρ| ≥ 0.60) that remained statistically significant after false discovery rate correction (FDR-adjusted q < 0.05) were retained for network analysis. We visualized these significant virus–virus correlations as an interaction network using NetworkX (Hagberg et al., 2008) in Python. In the resulting graph, nodes represent viruses (by their abbreviations) and edges connect virus pairs with positive or negative correlations exceeding the threshold, with edge thickness proportional to |ρ|. Because HTS libraries were generated from pooled leaves of multiple plants per region, the correlation analysis represents regional-scale co-occurrence patterns rather than direct plant-level co-infection.

### Hierarchical clustering and ordination analyses

To examine regional variation in virus community composition, we applied hierarchical clustering and principal component analysis (PCA) to normalized abundance data. Virus relative abundances were log_10_(RPKM + 1) transformed. We generated a clustered heatmap using Python (Seaborn v0.12), employing Bray–Curtis distance and average linkage clustering for both the samples (columns) and viruses (rows). This approach visualized the similarities among regional viromes and highlights the main virus groups driving those patterns.

For PCA, the matrix of normalized virus abundance (RPKM) values across the nine libraries was used as input. Prior to ordination, values were log_10_(x + 1) transformed, centered, and scaled. PCA was performed using the scikit-learn package (Pedregosa et al., 2011).

## Results

### Virome composition and regional distribution

High-throughput sequencing of the nine pooled libraries yielded a comprehensive snapshot of the *C. officinale* virome. Note that each regional library represents a pooled signal from ≥5 plants (≥3 leaves per plant), with nucleic acids extracted per plant and then combined, so read abundances reflect composite community profiles at the regional scale. In total, 31 distinct viruses were infecting *C. officinale*, encompassing previously reported pathogens as well as several novel viruses. The total viral read counts and the presence/absence of each virus varied considerably across regions (**Table 1**). All libraries contained multiple viruses (co-infections), but the dominant viruses and their relative abundances differed by location. Sequencing depth and host-filtered read statistics for each library are shown in **Supplementary Figure S2**, confirming consistent library quality and sufficient viral read coverage across all regions.

Notably, a cytorhabdovirus-like sequence was detected (albeit at low abundance) in samples from BH-A, SC-A, PC, JA, and GC. BLASTn analysis showed it shared 77.89% nucleotide identity with glehnia littoralis virus 1 (NC_076977.1) and 70.25% identity with chelidonium alphacytorhabdovirus 1 (BK064268.1). Based on ICTV species demarcation criteria and its distinct phylogenetic placement, this virus was deemed a novel species. We tentatively named it cnidium officinale virus 1 (CnoV1).

Two unclassified tombusviridae-like sequences were consistently identified in samples from all nine regions. The first sequence showed <77.64% identity to arracacha latent virus E-associated RNA (ON603910.1) and 76.29% to plant tombusvirus-like associated RNA 1 (OL472298.1). The second sequence exhibited <83.09% identity to plant tombusvirus-like associated RNA 2 (OL472295.1) and 68.08% identity to saffron tombusvirus-like associated RNA (BK065167.1). Because the overall identities of both sequences to known viruses below 85%, we considered them novel species and tentatively named them cnidium virus Y and cnidium virus Z, respectively.

Additionally, we detected a satellite RNA-like sequence present in all libraries. BLASTn analysis showed <74.11% identity to peony yellowing associated secovirus satellite RNA (MN259157.1) and <74.56% identity to strawberry latent ringspot virus satellite RNA (AY860980.1). Based on these low similarities and its unique sequence characteristics, we classified this RNA as a new satellite species, provisionally named cnidium virus-associated satellite RNA (satCnV) (**Table 3**). The genome organizations of the four novel viruses are depicted in **Supplementary Figure S3**, highlighting canonical ORF arrangements consistent with cytorhabdovirus and tombusviridae-like genomes.

**Table 3:** BLAST results for novel viruses.

Detected virus	BLAST type	Scientific name	Max score	Total score	Query cover	E value	Per. ident	Acc. len	Accession
Cnidium officinale virus 1	BLASTn	Glehnia littoralis virus 1	9738	9738	98%	0.0	77.89%	12193	NC_076977.1
		Chelidonium alphacytorhabdovirus 1	2716	3812	68%	0.0	70.25%	12148	BK064268.1
Cnidium virus Y		Arracacha latent virus E associated RNA	1716	1716	79%	0.0	77.64%	2861	ON603910.1
		Plant tombusvirus-like associated RNA 1	1426	1562	77%	0.0	76.29%	2962	OL472298.1
Cnidium virus Z		Plant tombusvirus-like associated RNA 2	2499	2499	82%	0.0	83.09%	2505	OL472295.1
		Saffron tombusvirus-like associated RNA	451	451	48%	7e-121	68.08%	2649	BK065167.1
Cnidium virus-associated satellite RNA		Peony yellowing associated secovirus satellite RNA	490	490	79%	3e-133	74.11%	1092	MN259157.1
		Strawberry latent ringspot virus satellite RNA	141	141	22%	5e-28	74.56%	1117	AY860980.1

BLASTn analysis was conducted on four putative novel plant viral sequences identified from nine *Cnidium officinale* libraries. These included one sequence related to the genus *Cytorhabdovirus*, two unclassified *Tombusviridae*-like sequences, and one RNA satellite. All sequences showed significant homology to known viral sequences, supporting their identification.

**Figure 1:**
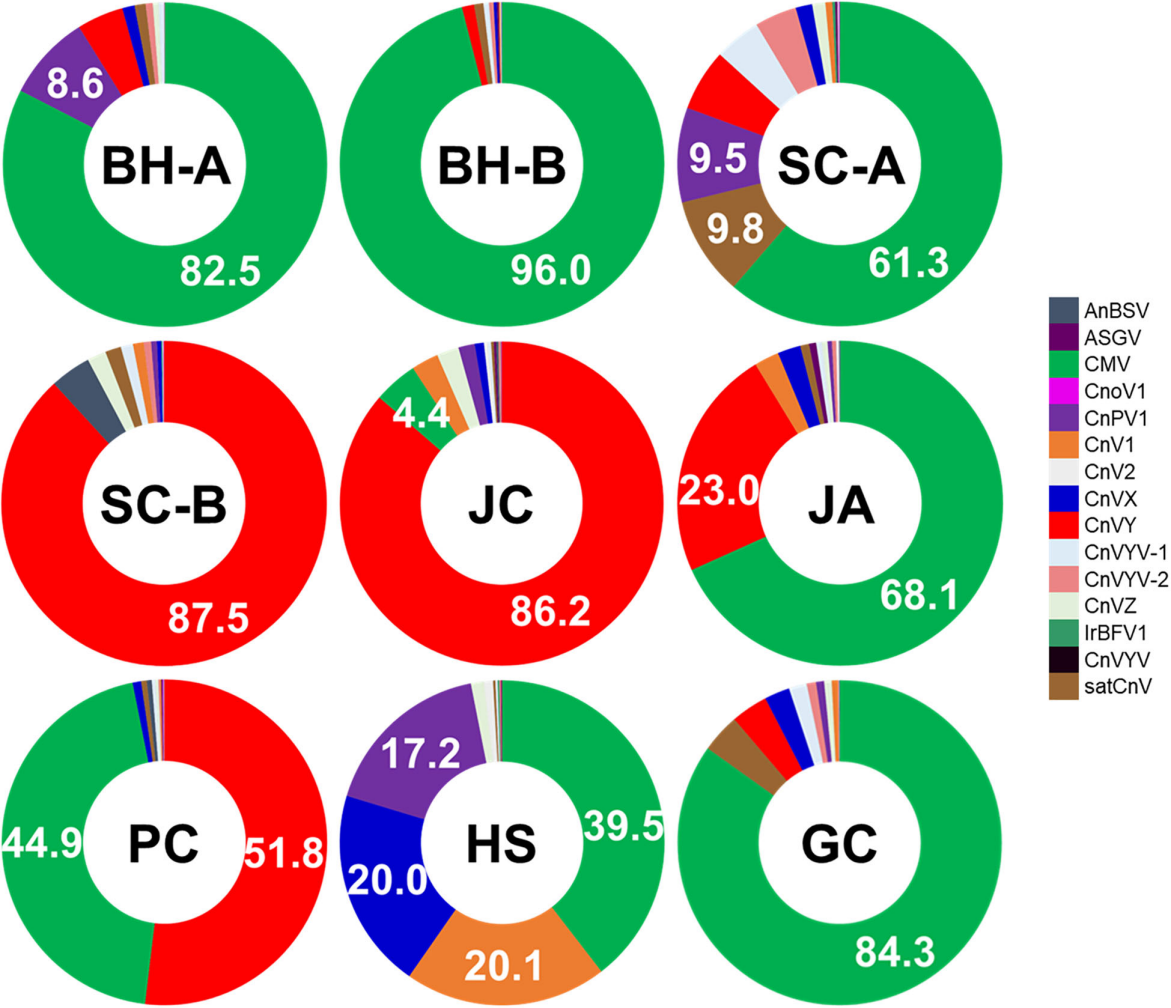
Proportion of viral‐derived reads in *Cnidium officinale* libraries from nine Korean regions. Each bar shows the percentage of reads mapping to viral genomes after host‐read filtering. Site abbreviations are defined as follows: BH-A, Myeongho-myeon (Bonghwa); BH-B, Socheon-myeon (Bonghwa); SC-A, Geundeok-myeon (Samcheok); SC-B, Hajang-myeon (Samcheok); HS, Hoengseong; GC, Geochang; JC, Jecheon; PC, Pyeongchang; and JA, Jinan. Percentages are indicated above each bar.

**Figure 2:**
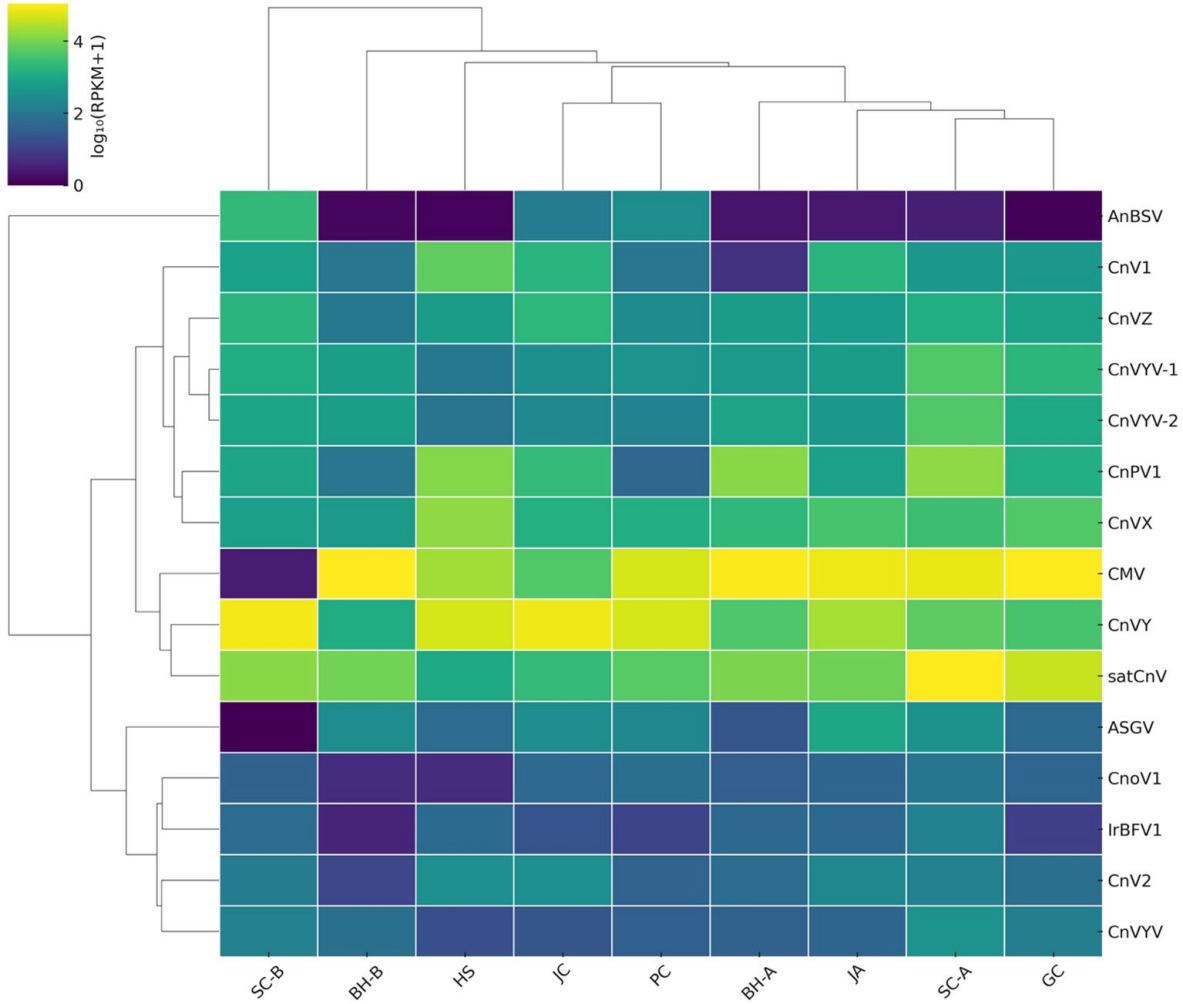
Heatmap illustrating the abundance of major viruses detected in *Cnidium officinale* samples from nine distinct cultivation regions in Korea. Data represent normalized abundance values calculated as log_10_(RPKM + 1). Hierarchical clustering was performed using average linkage with Bray–Curtis distance. Viruses (rows) are represented by abbreviations; regions (columns) indicate sampling sites.

For example, CMV and CnVX were detected in all nine regions, often comprising a large fraction of the viral reads in many samples. In Bonghwa (both BH-A and BH-B) and Geochang (GC), CMV was the overwhelmingly dominant virus, accounting for tens of millions of reads at those sites (**Figure 1**, **Table 1**). By contrast, in Samcheok-Hajang (SC-B) and Jecheon (JC), CMV reads were scarce; instead, the novel CnVY was the most abundant virus (exceeding 10^7^ reads in each of those libraries). In BH-A, BH-B, and GC, CnVY was present but at a lower relative read abundance than CMV (CnVY: CMV read ratios ≈ 0.06, 0.01, and 0.05, respectively). By contrast, both viruses were abundant in HS (CnVY = 35.7 M; CMV = 11.8 M) and PC (CnVY = 21.4 M; CMV = 16.9 M). Other viruses exhibited intermediate or region-specific patterns. For example, ASGV (a ubiquitous fruit tree virus) was found in all regions but at moderate read counts, whereas angelica bushy stunt virus (AnBSV) was extremely abundant in SC-B yet nearly absent in other sites. A clustered heatmap of virus abundance profiles (**Figure 2**) highlights these patterns. Hierarchical clustering based on Bray–Curtis similarity grouped together regions with similar dominant viruses. Notably, the two Bonghwa libraries (BH-A and BH-B) clustered with Geochang, reflecting their shared high CMV prevalence (and relatively lower CnVY levels). In contrast, the Samcheok-B (SC-B) and Jecheon (JC) libraries clustered together, both characterized by a predominance of CnVY and minimal CMV. Other sites like Hoengseong (HS) and Pyeongchang (PC) showed mixed profiles (both CMV and CnVY were abundant) and occupied intermediate positions in the cluster dendrograms.

**Figure 3:**
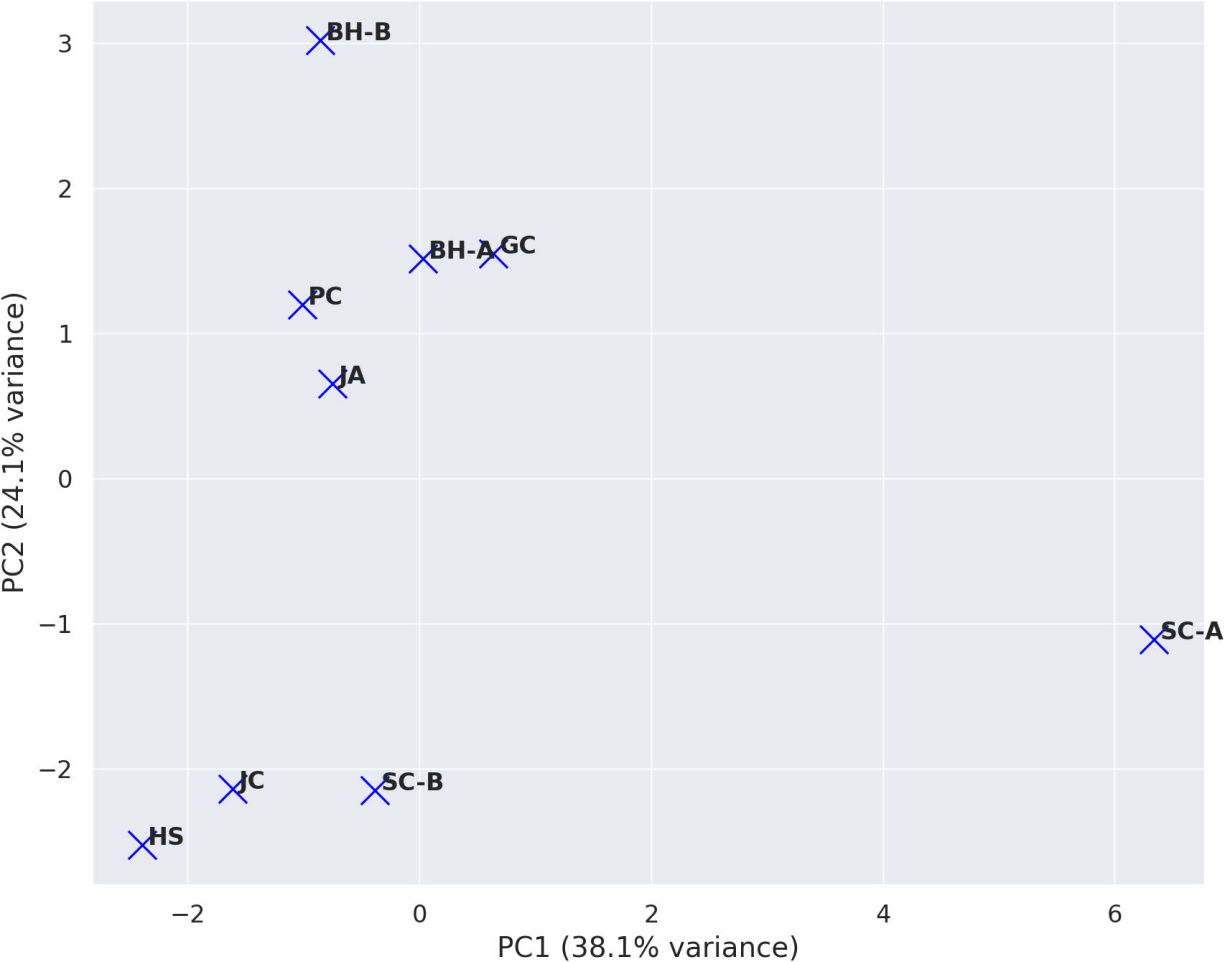
Principal component analysis (PCA) illustrating regional differences in virus community composition. PCA was performed based on normalized RPKM values of major viruses identified in *Cnidium officinale* samples across nine distinct cultivation regions in Korea. Each point represents a region, and proximity indicates similar virus compositions. The percentage of variance explained by each principal component is indicated in parentheses.

To further synthesize regional differences in virome composition, we applied both clustering and principal component analysis (**Figures 2**, **3**). A clustered heatmap of log_10_(RPKM + 1) values (**Figure 2**) delineated three broad community types: (i) CnVY-rich libraries (notably SC-B, JC, and HS), (ii) CMV-dominant libraries (BH-A/B, PC, and JA), and (iii) CnVYV–satCnV-rich libraries (SC-A and GC). These clusters align with the per-region read distributions (**Table 1**) and indicate that CnVY, CMV, and the CnVYV–satCnV complex define the major axes of virome variation in *C. officinale*.

Principal component analysis (**Figure 3**) provided a complementary view of these patterns. PC1 (accounting for 38.1% of variance) separated the CnVY-rich libraries (left side of the plot: SC-B, JC, HS) from the CnVYV–satCnV-rich libraries (right side: SC-A, GC). PC2 (24.1% of variance) largely reflected the relative abundance of CMV, distinguishing CMV-dominant regions (BH-A/B, PC, JA) from those with lower CMV levels (SC-B, JC). Although HS contained substantial CMV reads, its low PC2 score resulted from the greater contributions of CnVY and CnV1 in that library. Overall, the PCA patterns corroborate the clustered heatmap results, underscoring that regional virome variation is shaped by the balance between CnVY, CMV, and the CnVYV–satCnV complex.

### Virus co-infection network and correlation analysis

Given the high incidence of mixed infections, we next examined associations among co-infecting viruses. A Spearman rank correlation analysis of virus RPKM across the nine libraries revealed several significant pairwise relationships (|ρ| ≥ 0.60, FDR-adjusted q < 0.05), summarized in **Table 4**. These significant correlations are visualized as a virus co-occurrence network in **Supplementary Figure S4**. The network reveals a tightly interconnected cluster of positive correlations involving the cnidium vein yellowing virus complex (CnVYV-1 and -2) and the satellite RNA. Specifically, CnVYV-1 and CnVYV-2 were very strongly correlated with each other (ρ ≈ 0.93), as expected since both segments derive from the same bipartite virus and consistently co-occur in infected plants. Moreover, each of these viruses showed a strong positive correlation with the novel satellite RNA satCnV (ρ ranging from 0.95 to 0.98 with CnVYV-1 and CnVYV-2). This indicates that the satellite was almost always present when the CnVYV complex was present. Such tight linkage suggests that satCnV is specifically associated with CnVYV complex infections, possibly hitchhiking on the CnVYV complex as a satellite that utilizes those viruses as helpers.

**Table 4:** Summary of significant pairwise virus-virus correlations calculated using Spearman’s rank correlation.

Virus 1	Virus 2	Spearman correlation (ρ)	Raw P-value	FDR-adjusted Q-value
CnVYV-1	CnVYV-2	0.93	2.36e-04	5.73e-04
CnVYV-1	satCnV	0.95	8.76e-05	3.72e-04
CnVYV-1	CnVYV	0.98	1.94e-06	1.10e-05
CnVYV-2	satCnV	0.98	1.94e-06	1.10e-05
CnVYV-2	CnVYV	0.92	5.07e-04	1.08e-03
CnV1	CnV2	0.93	2.36e-04	5.73e-04
CnVY	CMV	-0.98	1.94e-06	1.10e-05
satCnV	CnVYV	0.93	2.36e-04	5.73e-04

Only significant correlations (|ρ| ≥ 0.60, FDR-adjusted q-value < 0.05) are shown. Columns indicate virus pairs (abbreviated names), Spearman correlation coefficient (ρ), raw P-value, and FDR-adjusted q-value.

In addition to the CnVYV complex-satCnV cluster, the co-occurrence network identified other notable associations. We found a robust positive correlation between two novel viruses, CnV1 and CnV2 (ρ ≈ 0.93, *q* < 0.001). Their strong co-occurrence suggests these viruses might share a common ecological or epidemiological link, such as being transmitted by the same vector or thriving under similar host conditions. It is also possible that one facilitates infection by the other, although further experiments would be needed to test any direct interactions.

In contrast, we observed a strong negative correlation between CnVY (the novel cnidium virus Y) and CMV (ρ ≈ –0.98, *q* < 0.0001). In samples where CMV was extremely abundant (e.g., the Bonghwa and Geochang libraries), CnVY was present at only low levels or not at all. Conversely, in libraries where CnVY dominated (Samcheok-B and Jecheon), CMV was virtually absent. This inverse relationship suggests an antagonistic interaction or mutual exclusion between CMV and CnVY in *C. officinale*. One virus may competitively suppress the other, or they may each thrive under different conditions (for example, differing vector presence or timing of infection). Another possibility is that cultivation history (exchange of infected planting stock) caused certain regions to end up with predominantly one virus or the other. This scenario would manifest as the strong negative correlation observed in our data.

### Phylogenetic relationships of major viruses

**Figure 4:**
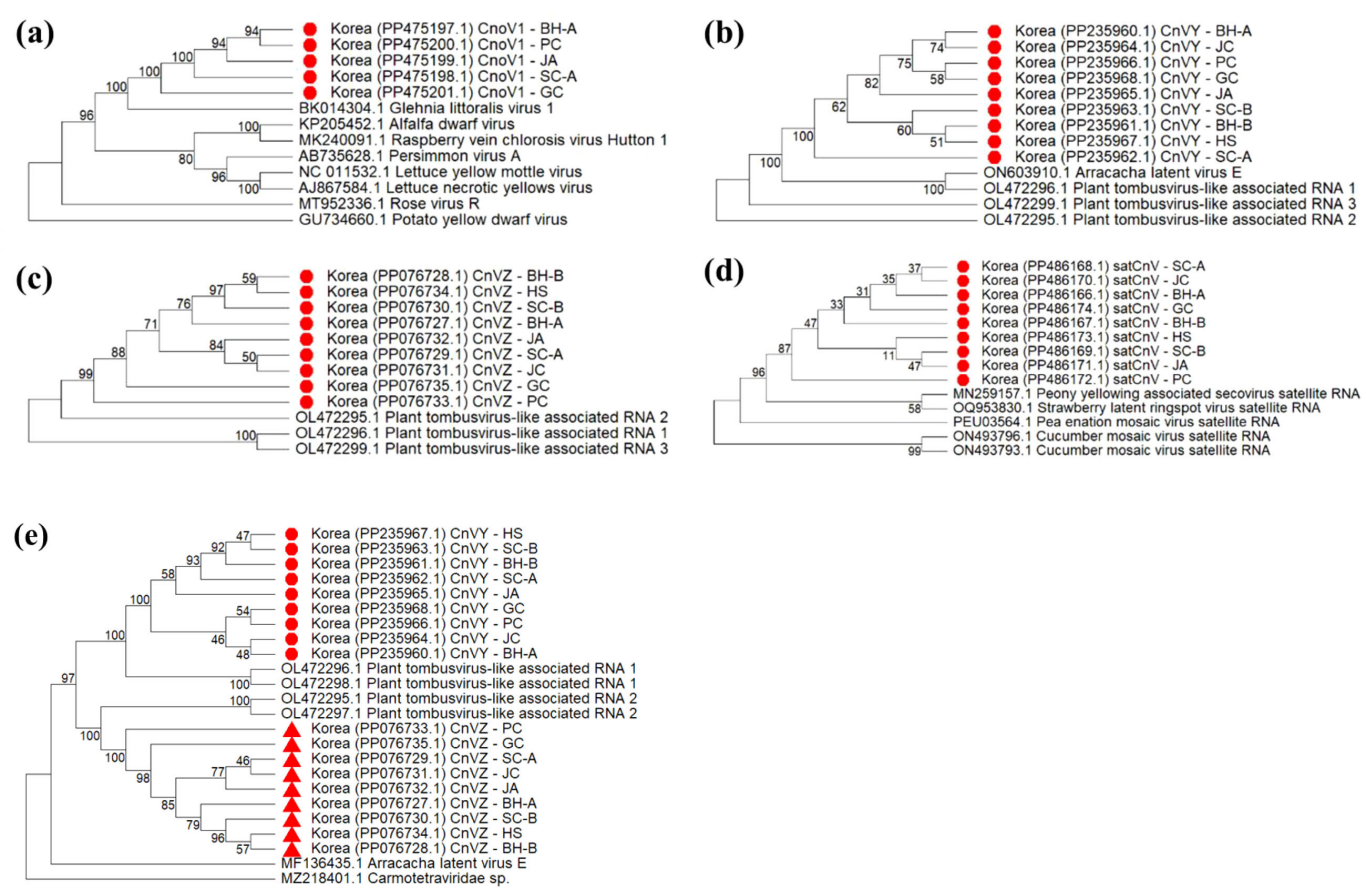
Maximum‐likelihood phylogenetic trees of novel viruses detected in *Cnidium officinale*. **(a)** Tree for five cnidium cytorhabdovirus 1 (CnoV1) isolates (BH-A, SC-A, PC, JA, GC) alongside reference *Rhabdoviridae* genomes. **(b)** Tree for nine cnidium vein‐yellowing virus isolates (CnVY and CnVZ; sites BH-A, BH-B, SC-A, SC-B, JC, JA, PC, HS, GC) with *Tombusviridae* genus references. **(c)** Tree showing satellite RNA (satCnV) sequences along with known *Secoviridae* satellite RNAs. All trees were inferred using MEGA11 with the neighbor‐joining method and 1,000 bootstrap replicates; bootstrap support ≥ 70% is indicated at the nodes, and branch lengths represent nucleotide substitutions per site. Korean isolates from this study are highlighted in red.

**Figure 5:**
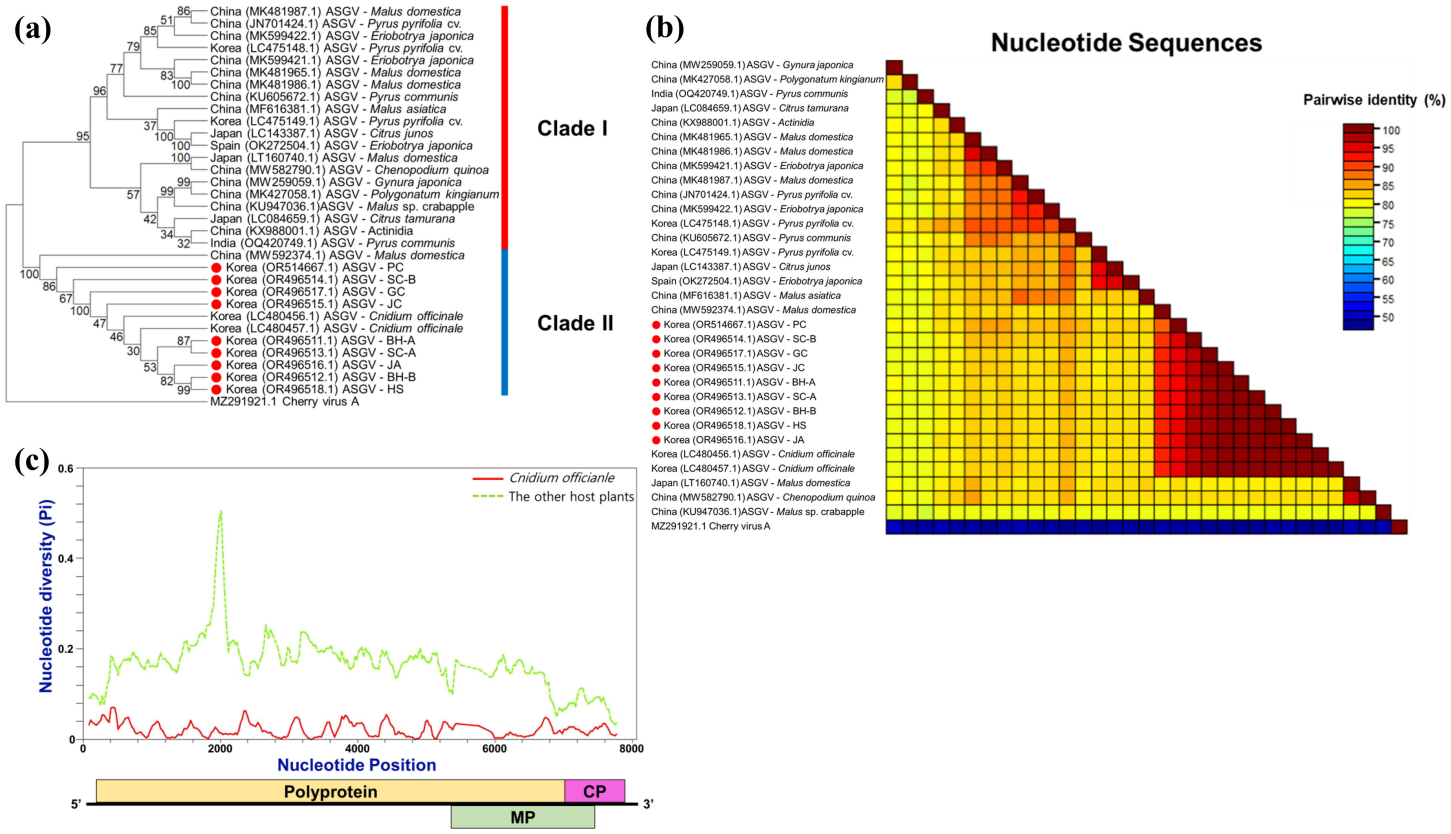
Phylogenetic relationships and nucleotide‐diversity profiles of ASGV isolates in *Cnidium officinale*. **(a)** Neighbor‐joining phylogenetic tree of complete ASGV genomes (isolates from this study plus reference sequences), inferred in MEGA11 with 1–000 bootstrap replicates; bootstrap values ≥ 70% are shown. **(b)** Pairwise percent‐identity matrix of ASGV coat‐protein sequences computed by SDT v1.3 (red = low identity/higher divergence; blue = high identity/lower divergence); reference strain names appear along axes. **(c)** Sliding‐window nucleotide diversity (π) across ASGV genomes (window = 200 nt, step = 50 nt) calculated in DnaSP v6; the x‐axis indicates genome position and the y‐axis shows π values for each alignment window.

To place the *C. officinale* viruses in an evolutionary context, we performed phylogenetic analyses for the predominant viruses. For brevity, we focus here on ASGV and CMV, while detailed phylogenies for other viruses are provided in **Figure 4**, **Supplementary Figures S5**, **S6**. A neighbor-joining tree for ASGV revealed that the *C. officinale* isolates from Korea cluster closely together in a clade distinct from ASGV isolates infecting other hosts (**Figure 5A**). This host-specific clustering suggests that the ASGV population in *C. officinale* has diverged from ASGV in other hosts, possibly due to adaptation to the *Cnidium* host or limited gene flow from outside populations. A pairwise nucleotide identity heatmap of ASGV coat protein sequences (**Figure 5B**) confirmed that the *C. officinale* isolates are nearly identical to each other (> 99% identity) but show much greater divergence from ASGV isolates in apple and other hosts. Consistently, nucleotide diversity (π) across the genomes of *C. officinale* ASGV was exceedingly low (10^–3^), and the diversity profile along the genome was relatively flat (**Figure 5C**), indicative of uniformly strong purifying selection.

**Figure 6:**
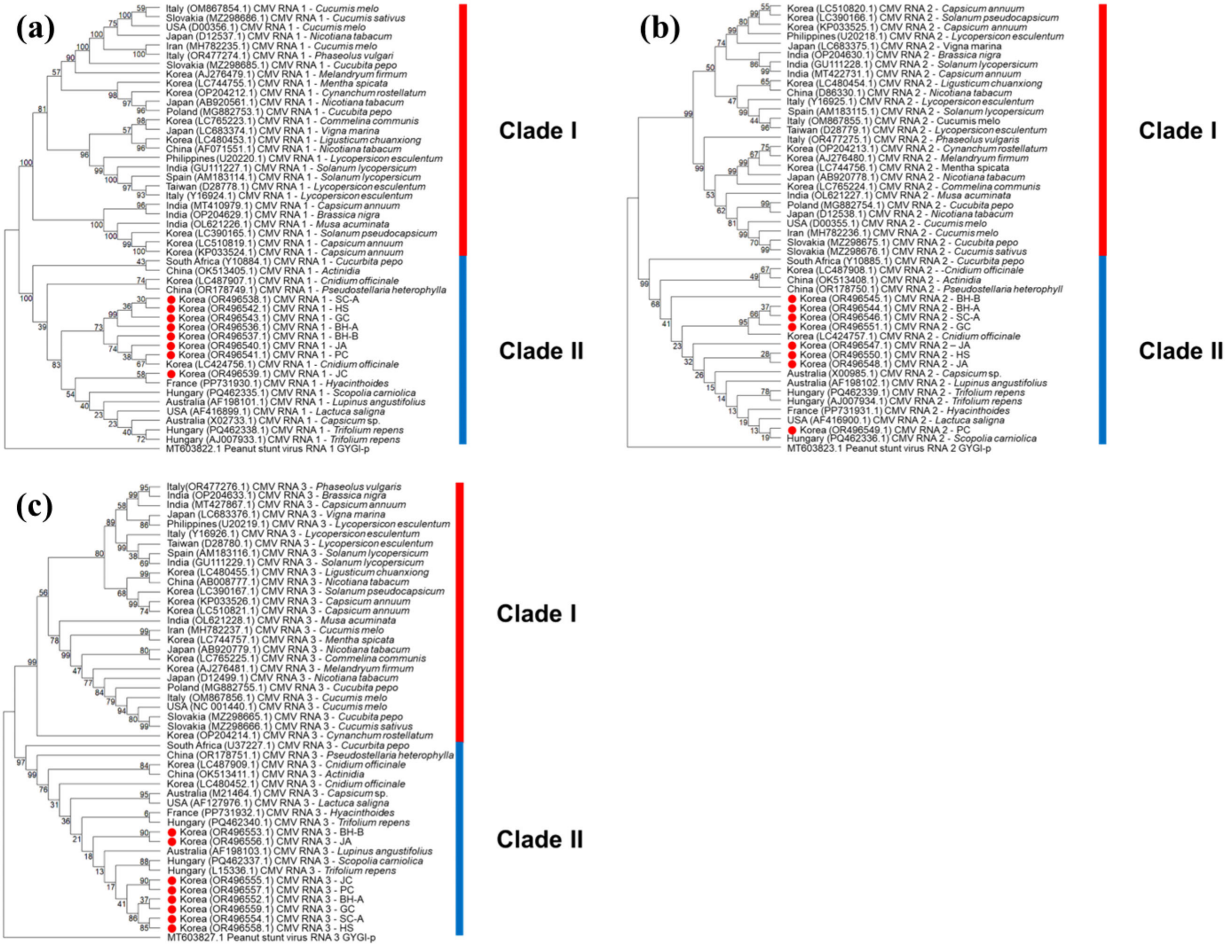
Neighbor‐joining phylogenies of CMV RNA segments from *Cnidium officinale* isolates. **(a)** CMV RNA 1, **(b)** CMV RNA 2, and **(c)** CMV RNA 3 complete genomes were aligned with reference sequences. Trees were inferred in MEGA11 using the neighbor‐joining method with 1,000 bootstrap replicates; bootstrap values ≥ 70% are displayed. Korean isolates from this study are marked in red, and branch lengths indicate nucleotide substitutions per site.

**Figure 7:**
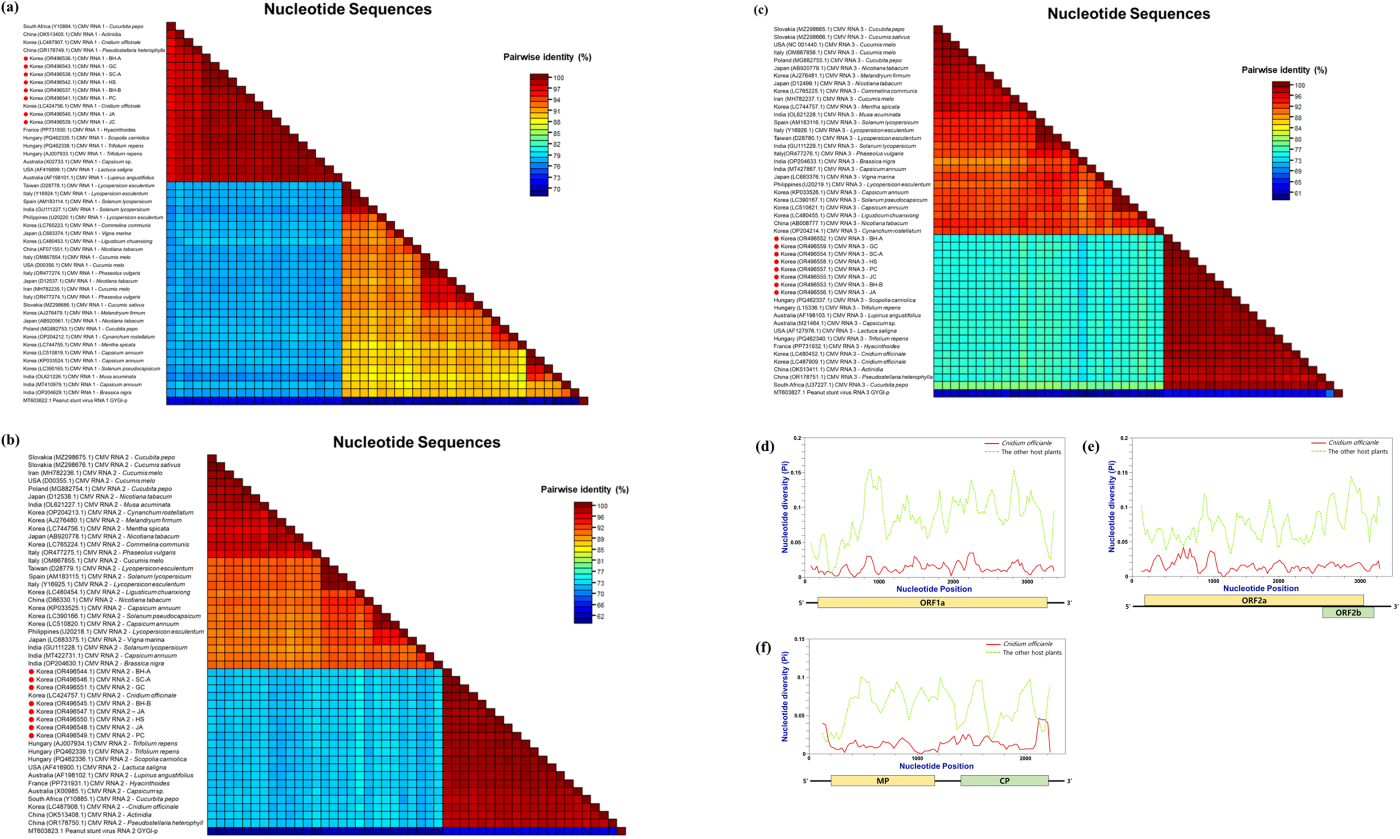
Pairwise identity matrices and sliding‐window nucleotide‐diversity profiles of CMV segments. **(a)** Pairwise percent‐identity matrix for CMV RNA 1 sequences (this study vs. references); **(b)** CMV RNA 2; **(c)** CMV RNA 3. In each matrix, cells in the upper triangle show percent identity (dark blue = high identity; light red = low identity), and cells in the lower triangle indicate percent divergence (mirror of the upper). **(d–f)** Sliding‐window nucleotide diversity (π) across **(d)** RNA 1, **(e)** RNA 2, and **(f)** RNA 3 alignments (window = 200 nt, step = 50 nt), calculated in DnaSP v6; the x‐axis is genome position, and the y‐axis is π. Reference isolate names appear on each axis, and Korean isolates are highlighted in bold.

CMV exhibited a similar pattern of host-specific uniformity. Complete genome sequences of CMV (RNAs 1–3) from *C. officinale* formed a monophyletic group within the subgroup IA lineage of CMV, distinct from most CMV isolates infecting other plant species (**Figure 6**). Despite originating from widely separated regions, the *C. officinale* CMV isolates showed minimal genetic variation among themselves. The average pairwise nucleotide identity among these nine isolates exceed 99.5% for each RNA segment, with only a handful of single-nucleotide polymorphisms distinguishing them. Sliding-window analyses of nucleotide diversity along each CMV genomic RNA (**Figures 7D–F**) showed uniformly low π values across all three RNAs, consistent with a recent common origin and/or strong purifying selection. Additionally, the *C. officinale* CMV isolates shared several signature mutations not observed in CMV isolates from other hosts, supporting the idea that these isolates constitute a host-adapted lineage.

## Discussion

In this study, we conducted a comprehensive HTS survey of viruses infecting *C. officinale* across nine major cultivation regions in Korea. This represents the first broad virome characterization for this medicinal, vegetatively propagated crop. We identified 31 distinct viruses, including several previously known plant viruses and four novel species (provisionally named CnoV1, CnVY, CnVZ, and the satellite RNA satCnV). The application of HTS enabled not only the discovery and cataloging of these viruses, but also an analysis of their relative abundances and co-infection patterns across different regions. These findings significantly expand our understanding of virus diversity in *C. officinale* and provide new insights into virus community ecology in clonally propagated medicinal crops.

Notably, the dominant viruses infecting *C. officinale* – including CMV, CnVYV (a vein-yellowing virus complex with two species), ASGV, and CnVX – exhibited remarkably high genetic uniformity across all surveyed regions. Sequence variants from geographically distant provinces were often nearly identical, and phylogenetic analyses consistently clustered isolates by host species (i.e., *C. officinale*) rather than by location. For example, CMV and ASGV sequences from *C. officinale* formed host-specific clades distinct from isolates of the same viruses infecting other host plants (**Figures 5**-**7**). The extremely low genetic diversity observed for viruses like CMV and ASGV in *C. officinale* suggests a long-term, closed association with this host. All sampled isolates of these viruses were nearly identical across widely separated provinces. They were also highly similar to one another but distinct from strains found in other crops in Korea. This implies very limited gene flow between *C. officinale* virus populations and those in other host species. Once a virus was introduced into *C. officinale*, it likely became genetically isolated and was maintained almost exclusively within *C. officinale* through vegetative propagation. Such host-restricted virus lineages have been noted in other systems and are thought to arise when viruses persist in a crop over long periods without frequent reintroduction from outside sources (Kawakubo et al., 2023). Our data therefore point to a pattern of long-term, stable maintenance of viruses in *C. officinale* with minimal diversification, most likely as a consequence of the plant’s clonal propagation habit (which limits opportunities for new viral genotypes to be introduced or selected). This situation contrasts with seed-propagated or more widely exchanged crops, where viral populations often show greater regional genetic differentiation. For instance, a virome study of sweet potato (another vegetatively propagated crop) found that while multiple viruses are ubiquitous, many virus isolates (especially potyviruses) exhibited grouping by geographical region (Jo et al., 2020). In *C. officinale*, by contrast, we did not observe region-specific virus clades – an indication that *C. officinale* viruses have been circulating largely within a closed host population, with very limited new introductions or host shifts.

Beyond the major viruses, our survey uncovered additional viral elements that could influence disease outcomes. A particularly noteworthy discovery is a novel satellite RNA, designated satCnV, associated with the *C. officinale* virome. To our knowledge, this is the first report of a satellite RNA in viruses infecting *C. officinale*. Satellite RNAs are small sub-viral molecules that depend on a helper virus for replication and encapsidation. They are known to modulate disease severity and viral accumulation in various plant–virus systems (Palukaitis, 1988; Collmer and Howell, 1992; Hu et al., 2009; Cao et al., 2019). For example, satellite RNAs in other pathosystems (e.g., those associated with cucumber mosaic virus or bamboo mosaic virus) can attenuate or exacerbate symptom expression in the host (Lin and Lin, 2017). The discovery of satCnV in *C. officinale* raises intriguing questions about its biological role: it may interact with its helper virus (likely the CnVYV complex, as discussed below) to influence symptom development or virus multiplication in this crop. Determining whether satCnV ameliorates or aggravates disease symptoms, and how it affects the epidemiology of its helper virus, will be an important subject for future research.

Our analysis of co-infection patterns revealed both antagonistic and synergistic relationships among the *C. officinale* viruses. In particular, we observed a strong inverse correlation between the abundance of CnVY (the single-segment vein-yellowing virus) and CMV across the pooled regional samples. At the regional level, libraries where CMV was extremely abundant tended to have lower representation of CnVY, and vice versa. For instance, CMV dominated the virome in Bonghwa and Geochang, whereas CnVY was prevalent in regions like Samcheok-B and Jecheon. It is important to note that this negative correlation reflects opposite abundance trends in pooled samples, rather than an absolute mutual exclusion within individual plants. In fact, both viruses were detected (often together) in all regions, and in some areas (e.g., Hoengseong and Pyeongchang) they were simultaneously abundant. Thus, their relationship appears to be a broad ecological antagonism rather than a strict within-plant incompatibility. One plausible explanation is competitive interactions or cross-protection: infection by one virus might suppress superinfection by the other, especially if one induces a strong RNA silencing response or occupies the available cellular “niche” in the host (Hanssen and Thomma, 2010; Folimonova, 2012; Boezen et al., 2023). Cross-protection is a known phenomenon where a plant infected by a mild strain of a virus is protected against a more severe strain of a related virus (Mascia and Gallitelli, 2016). In our case, if CMV and CnVY induce cross-reactive defense responses or compete for the same host resources, an increase in one could broadly limit the other. Another contributing factor could be differences in their insect vectors or transmission efficiency: if management or environmental factors in certain regions suppress the vector of one virus, the other virus (with a different vector) might gain a relative advantage. In any event, the regional-scale antagonism between CMV and CnVY cautions that controlling one virus in the field could inadvertently create ecological space for the other to proliferate. This interplay underscores the complexity of virus–virus interactions in mixed infections, which can significantly impact disease outcomes (Syller, 2012; Mascia and Gallitelli, 2016).

In contrast to the CMV–CnVY inverse relationship, we found a tightly linked cluster of positive correlations among the CnVYV-1 and CnVYV-2 segments (the two components of the vein-yellowing virus complex) and the satellite satCnV. Unsurprisingly, the two genomic segments of CnVYV co-occurred consistently, as both are required for a successful infection by this bipartite virus. More interestingly, the satellite RNA was almost always present alongside CnVYV, with read counts of satCnV closely tracking those of CnVYV-1 and CnVYV-2 in our regional pools. This suggests that satCnV is dependent on CnVYV as its helper virus and may have a functional association with that viral complex. The strong tripartite correlation hints that we are observing a stable satellite–helper virus partnership in *C. officinale*. It will be fascinating to investigate how satCnV influences the biology of CnVYV infections. In other well-studied systems, satellites can attenuate the damage of their helper viruses or, conversely, exacerbate symptoms, depending on the specifics of their interaction (Palukaitis, 1988; Collmer and Howell, 1992; Hu et al., 2009; Roossinck, 2015). For CnVYV and satCnV, experiments under controlled conditions (e.g., co-inoculating plants with CnVYV with and without the satellite) could elucidate whether satCnV mitigates symptom severity or perhaps impacts the transmission or persistence of the virus. The discovery of this satellite therefore opens a new line of inquiry into the pathogenicity and epidemiology of the vein-yellowing disease in *C. officinale*.

A striking aspect of our findings is the minimal regional differentiation in virus composition and sequence variants, which we attribute to *C. officinale*’s propagation practices. Because this crop is propagated almost exclusively by dividing rhizomes (clonal propagation), viruses are readily passed from mother plants to all vegetative progeny. Over years of cultivation, a region’s virome is likely shaped by whatever viruses were present in the initial planting stock (seed rhizomes) introduced to local farms, followed by amplification of those viruses through clonal spread within that region. In essence, each region’s virus community may represent a founder effect of the viruses present in the source propagative material, with relatively little influx of new viruses unless growers exchange plant material across regions (Delatte et al., 2007; Sun et al., 2013; Razavi et al., 2025). Consistent with this, our data show that the same few viruses (CMV, CnVYV, CnVY, CnVX, ASGV, etc.) dominate across all areas, and their sequences are nearly identical countrywide. This pattern is best explained by propagation-driven epidemiology rather than independent viral invasions in each locale. If farmers in different provinces originally obtained their *C. officinale* rhizomes from one or a few common nurseries (or from each other), those source plants would have “seeded” the observed regional viromes. Thereafter, local vegetative multiplication of infected stock would ensure the perpetuation of the same viruses in each clonal lineage. The strong host-specific clade formation discussed above is a natural consequence of this closed transmission loop. Indeed, vegetative propagation is known to facilitate long-term virus maintenance and can lead to an accumulation of viruses in clonally propagated crops. Sweet potato is an illustrative example: nearly all sweet potato plants in the field carry multiple viruses, largely because cuttings from infected mother plants disseminate viruses perpetually, and the lack of regular seed propagation prevents virome reset. Similarly, in *C. officinale*, the vegetative spread likely allows viruses to persist indefinitely once established, creating a relatively static virome within the crop.

Alternative factors such as vector-mediated transmission or differences in crop management could also influence regional virus prevalence, but we suspect these play a secondary role in this case. *C. officinale* is not known for extensive long-distance vector spread of its viruses (unlike, say, sweet potato where whitefly and aphids contribute to virus dissemination (Jo et al., 2020)). The near-uniformity of virus sequences across distant farms suggests that recurrent reintroductions or active vector-driven mixing between regions have been very limited. If vectors were regularly moving viruses between *C. officinale* fields or from other host species into *C. officinale*, we would expect more genetic variation or region-specific strains, which we did not observe. Therefore, we infer that clonal propagation is the dominant force structuring the virome of this crop, in line with what has been proposed for other vegetatively propagated plants (Gibbs et al., 2017; Jones and Naidu, 2019). Our conclusions on propagation-driven virome structure are necessarily somewhat indirect because our sampling did not include tracing of planting material sources. Future studies should explicitly examine the infection status of *C. officinale* propagation stocks—such as nursery rhizomes or “seed” bulbs—and track their distribution routes. If, for instance, a given nursery’s rhizomes are found to carry a specific set of viruses, and those viruses match the profiles in client farms across a region, it would strongly corroborate the idea that the regional viromes were seeded via that infected planting stock. Additionally, surveying potential insect vectors in these regions and testing whether they carry *C. officinale* viruses could help clarify the role of horizontal (vector-borne) spread. However, given our current data, the most parsimonious explanation is that the virome population structure is largely clonal, reflecting historical propagation practices with limited inter-regional exchange of infected plant material.

The findings of this virome study have direct practical implications for improving the health and quality of *C. officinale* crops. We identified a suite of persistent viruses that can infect *C. officinale*, many of which (like CMV, CnVYV, ASGV, etc.) are capable of causing latent or chronic infections. In a vegetatively propagated host, an infection is essentially permanent: once a mother plant is virus-infected, all of its clonal offspring will carry that virus load. This highlights the critical importance of establishing virus-free propagation programs. Similar “clean seed” or clean stock initiatives in other crops have been highly successful in curbing virus diseases – for example, certified virus-free seed potato programs and sweet potato virus indexing schemes have dramatically reduced yield losses caused by viruses (Gergerich et al., 2015). HTS-based virome analysis, as we have demonstrated, can guide the development of such programs by revealing which viruses are prevalent and must be targeted. In fact, there is a growing recognition that virome indexing should be integrated into plant biosecurity and seed systems, especially for vegetatively propagated crops (Alcalá Briseño et al., 2023). Clean germplasm production relies on sensitive detection of viruses in source plants, and our comprehensive list of *C. officinale* viruses now enables the design of molecular diagnostic tools (such as RT-PCR or RT-LAMP assays) for each major pathogen. We therefore urge the implementation of a virus-free certification program in *C. officinale* cultivation. In such a program, candidate mother stock plants would be rigorously tested for the presence of the key viruses identified in this study, and only those testing negative (virus-free) would be used to produce new rhizomes for distribution. By continuously indexing propagation material for viruses, growers can break the cycle of passing along infections and gradually purge the regional viromes from their fields.

To make virus indexing practical, we propose a two-tier diagnostic panel based on the viruses we found to be most widespread or economically significant. Tier 1 (core panel) would include the highest-priority targets for routine screening: CMV, CnVYV-1 and -2 (vein-yellowing virus segments), CnVY, CnVX, ASGV, Cnidium polerovirus 1 (CnPV1), and the satellite RNA satCnV. These agents were either ubiquitous across regions or associated with notable disease symptoms, making them crucial to detect and eliminate. Tier 2 (supplemental panel) would encompass additional viruses that might be included based on regional context or specific epidemiological concerns. This could include the novel viruses CnoV1, CnV1, CnV2, CnVZ (which were found in more limited instances), as well as other localized viruses such as Angelica bushy stunt virus (AnBSV) if relevant. In practice, a multiplex RT-PCR or a similar high-throughput assay could be developed to simultaneously test for all Tier 1 viruses in a single reaction per sample – an approach that many plant certification programs have employed successfully. Tier 2 agents can be tested in a follow-up manner or included in expanded panels when tracing back the source of an infection. By prioritizing the most common and damaging viruses for regular indexing, the workload remains feasible while still covering the majority of infection risks.

In implementing these recommendations, coordination among growers, nurseries, and plant protection authorities will be key. Growers should be educated about the long-term benefits of planting virus-free *C. officinale* rhizomes, even if that requires discarding some existing infected stocks. Meanwhile, nurseries or tissue-culture labs could be enlisted to maintain clean mother blocks of *C. officinale* – collections of plants that are tested and confirmed free of the Tier 1 viruses – from which propagation materials are multiplied under protected conditions (e.g., insect-proof greenhouses). Such a system would mirror the clean plant programs in horticultural industries like fruit trees and sweet potatoes. Over time, the availability of certified virus-free rhizomes would allow farmers to replace their diseased planting material, leading to healthier crops with higher yields and more consistent medicinal quality. Our virome dataset provides the foundational knowledge to initiate this process, as we now know exactly which pathogens to screen for. In a broader sense, this study exemplifies how HTS-driven virome analysis can be translated into practical strategies for crop improvement. By revealing the hidden burden of viruses in clonally propagated crops and pinpointing the culprits, we can design informed interventions to break the cycle of infection (Alcalá Briseño et al., 2023).

## Data Availability

The datasets presented in this study, including raw sequence reads and assembled viral genomes, are available in the NCBI Sequence Read Archive (SRA) under BioProject accession number PRJNA1031353. All novel virus genome sequences have been deposited in GenBank (see **Supplementary Table S2** for accession numbers). Additional supporting data (primer sequences, alignments, and phylogenetic trees) are provided in the Supplementary Material.
