# Supplementary material for: High-throughput virome profiling reveals complex viral diversity, co-infection patterns, and novel viruses in Cnidium officinale in Korea
Source: Front Plant Sci. 2025 Dec 10;16:1644750. doi: 10.3389/fpls.2025.1644750 (PMC12727994; doi:10.3389/fpls.2025.1644750)
Supplement: Supplementary file 1 [file Supplementaryfile1.docx]

**Supplementary Fig. S1**


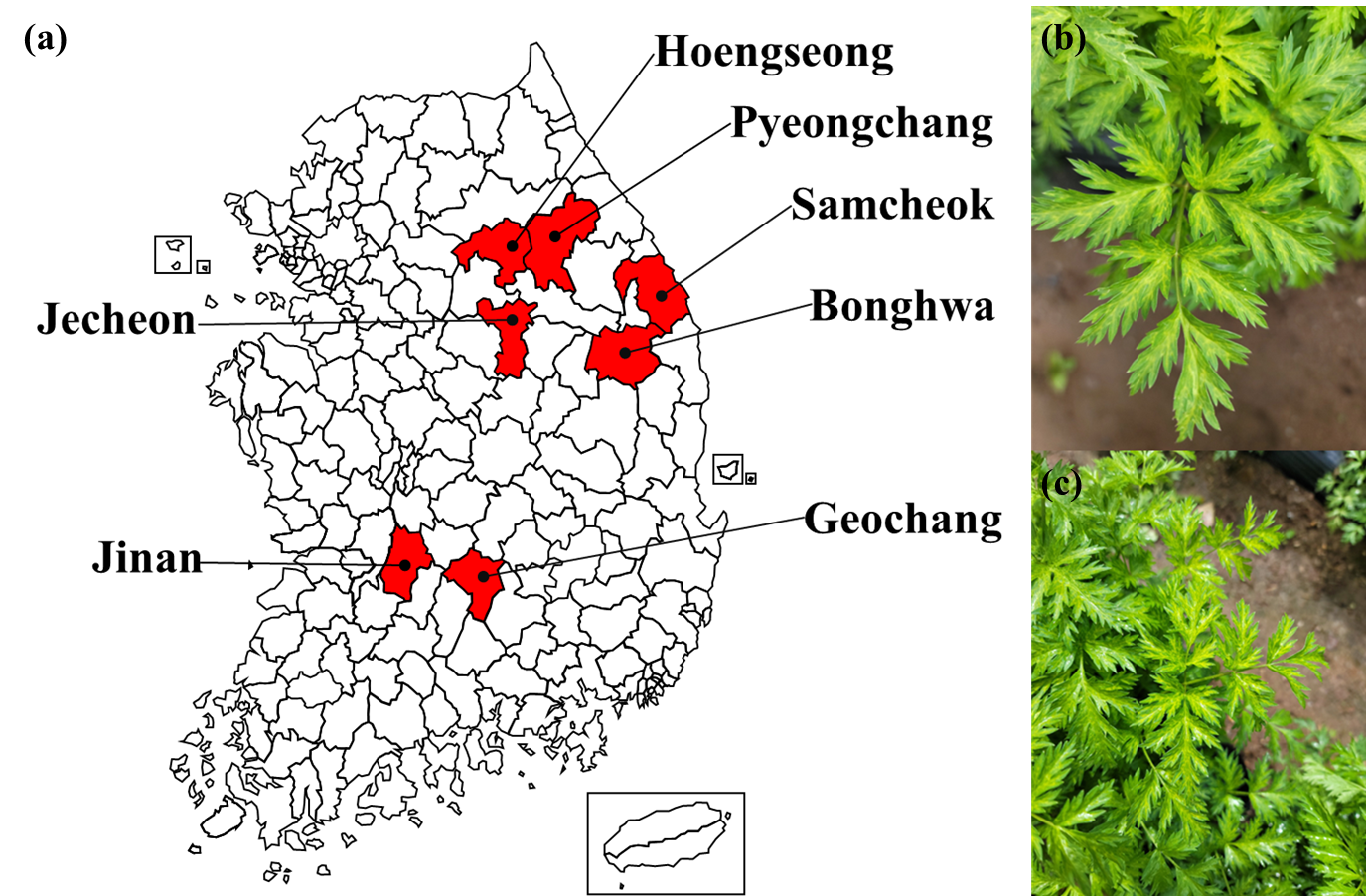


**Supplementary Fig. S1** Sample collection sites and representative symptoms of *Cnidium officinale*. **(a)** Geographic distribution of the nine sampling sites in Korea (red circles). **(b–c)** Plants showing vein-yellowing and chlorosis at Samcheok-B and Jecheon, respectively.

**Supplementary Fig. S2**


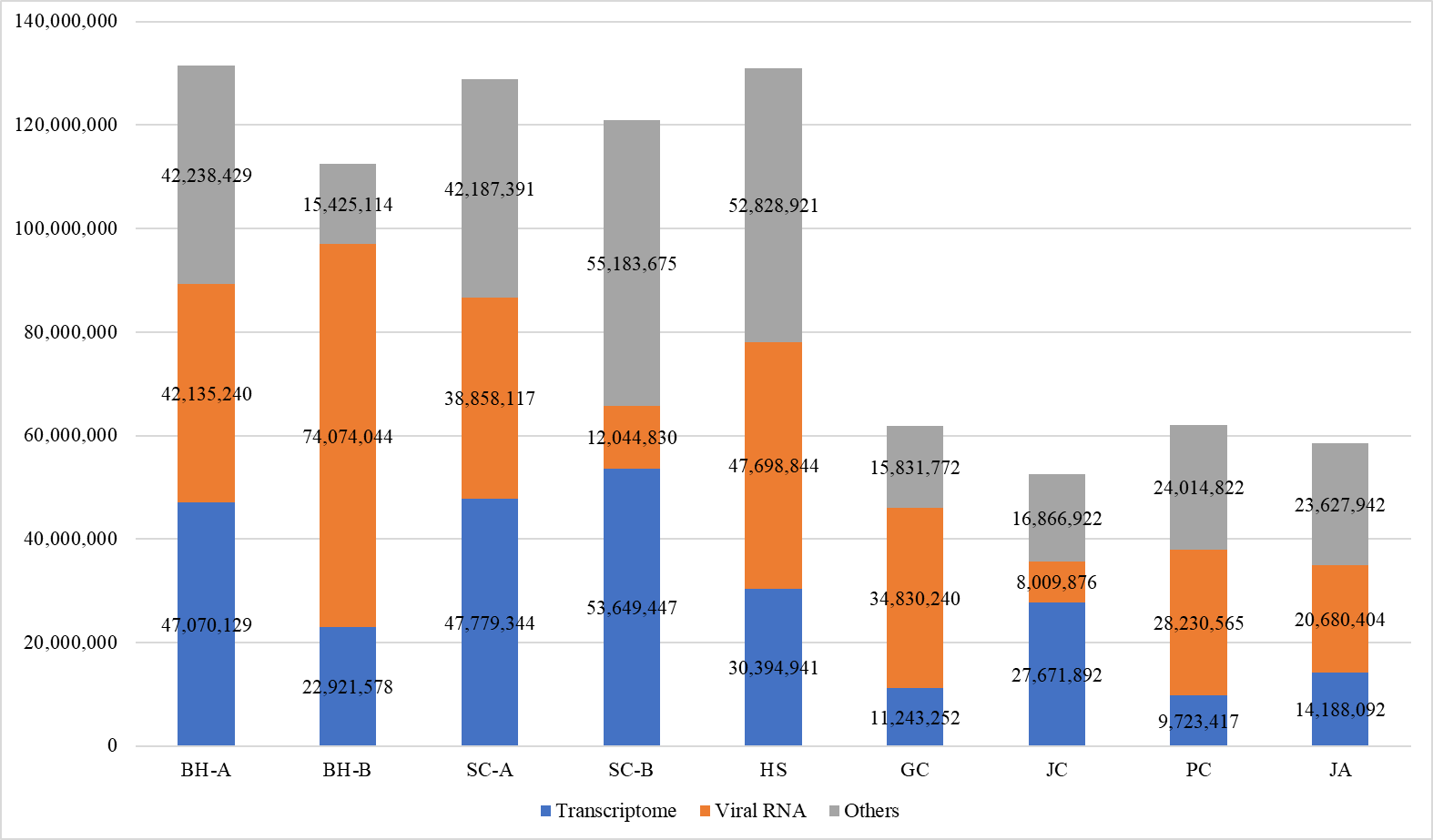


**Supplementary Fig. S2** Total number of reads per library. Bars represent the absolute count of reads that aligned to host transcriptomic (blue) and viral contigs (Orange).

**Supplementary Fig. S3**


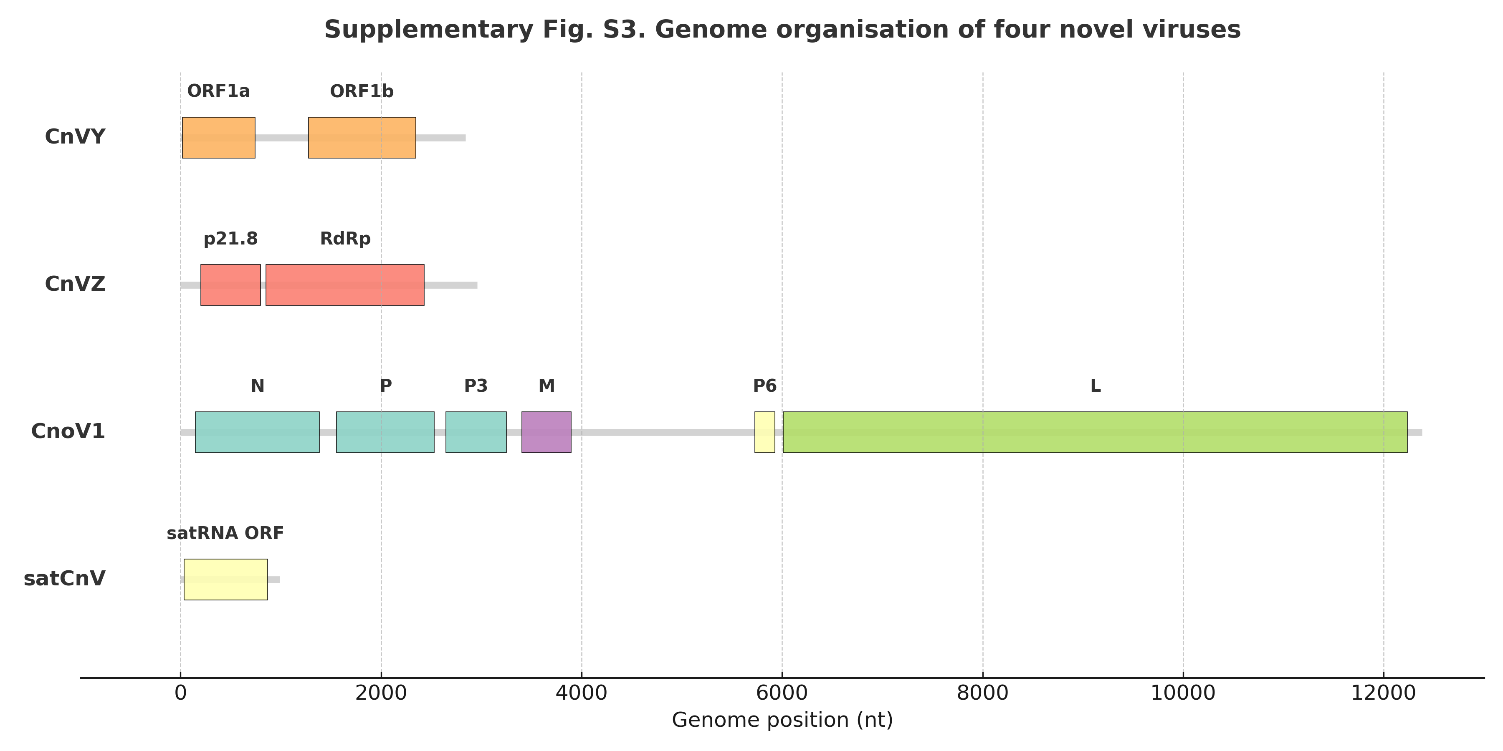


**Supplementary Fig. S3** Genome organization of the four novel viruses detected in *C. officinale*. Open reading frames are drawn to scale for CnVY, CnVZ, CnoV1 and satCnV, based on the GenBank annotations listed in Supplementary Table S2.

**Supplementary Fig. S4**


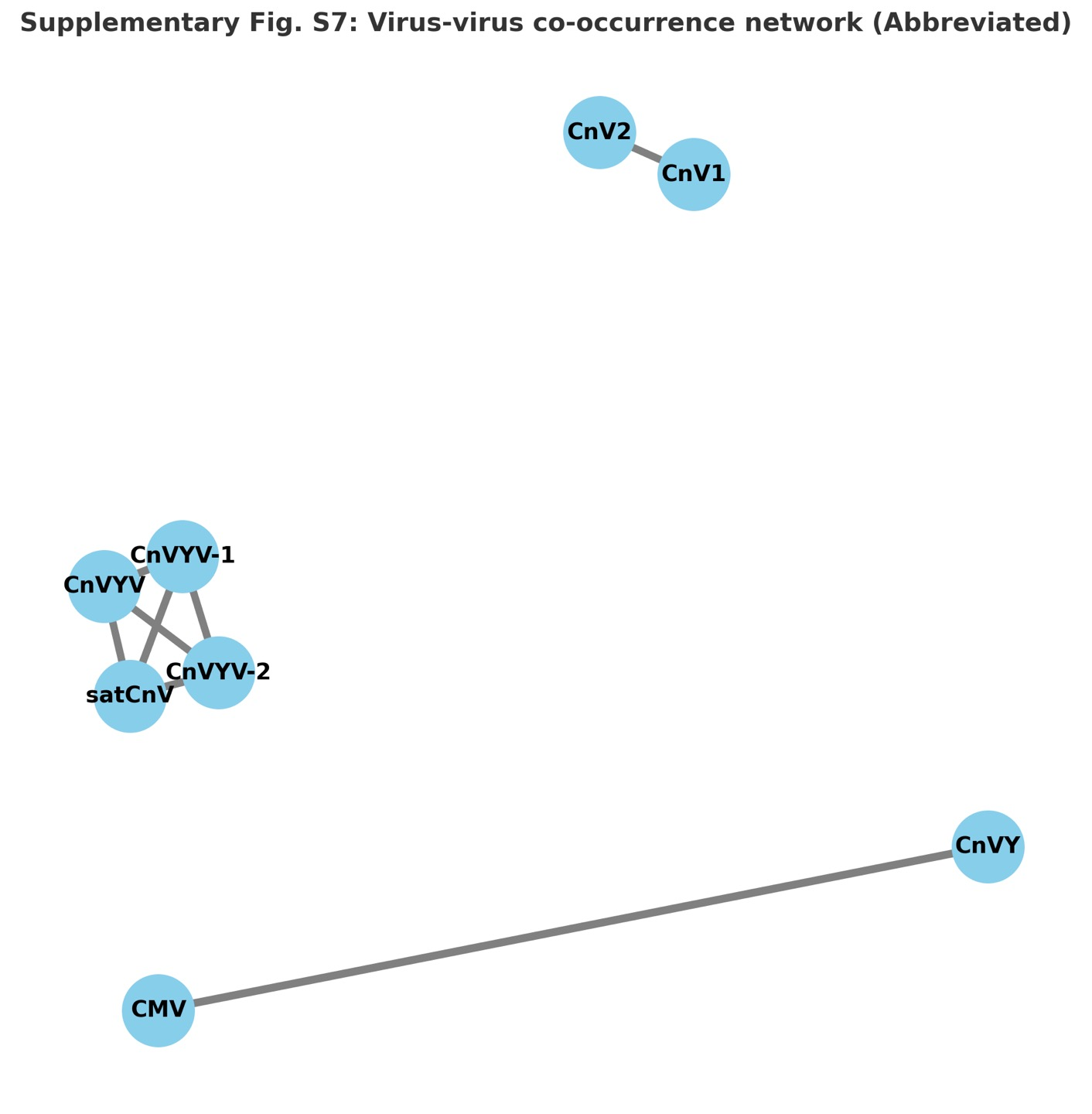


**Supplementary Fig. S4** Virus-virus co-occurrence network constructed based on significant pairwise Spearman rank correlations (|ρ| ≥ 0.60, FDR-adjusted q-value < 0.05). Nodes represent viruses (abbreviated names), and edges indicate significant correlations, with edge width proportional to the correlation strength. Positive and negative correlations are illustrated uniformly. .

**Supplementary Fig. S5**


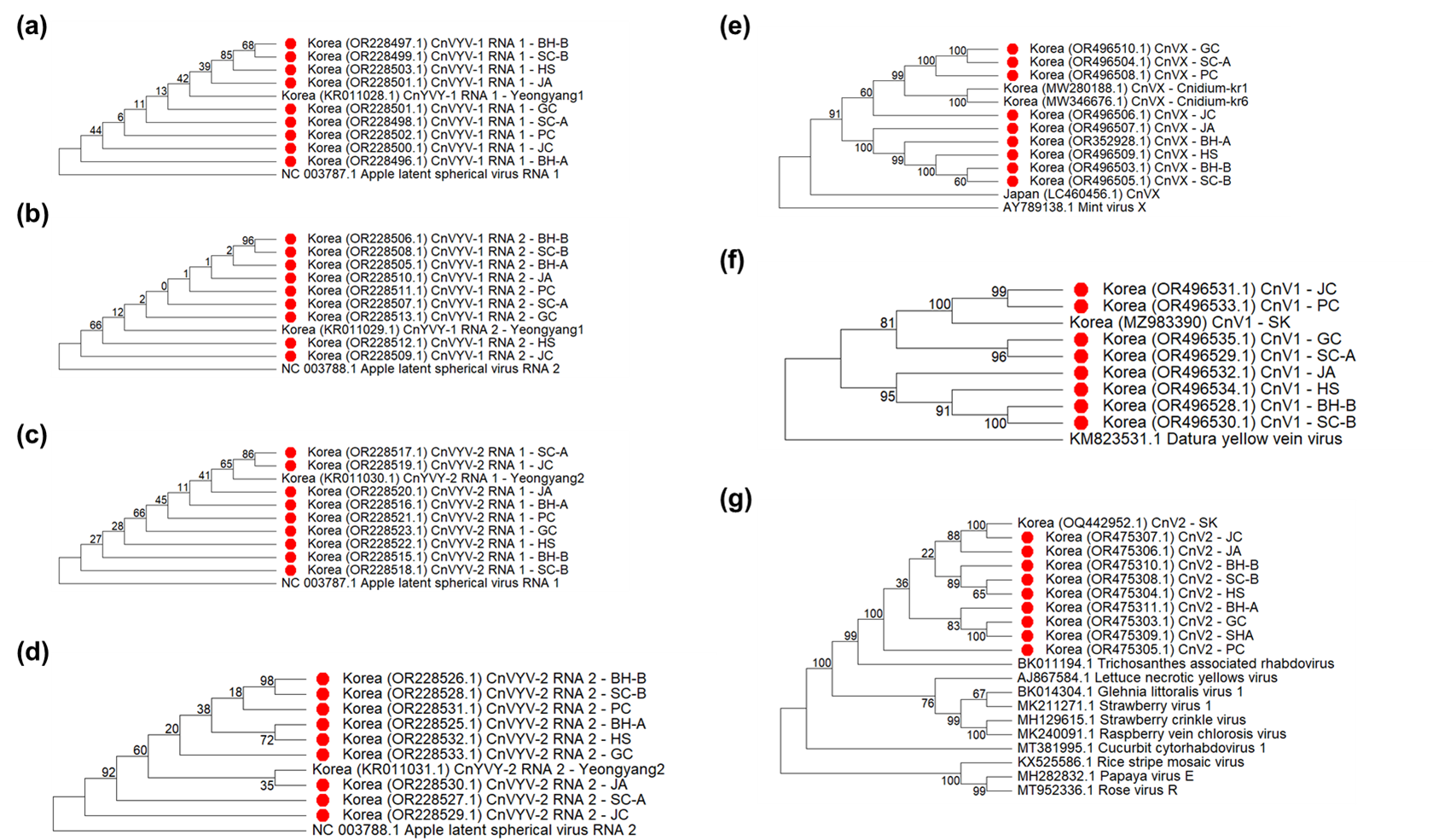


**Supplementary Fig. S5** Phylogenetic analysis of tombusvirus-like and unclassified cnidium viruses. Maximum-likelihood trees (1,000 bootstrap replicates) were inferred from complete or near-complete RNA-dependent RNA polymerase (RdRp) nucleotide sequences. **(a)** Cnidium vein-yellowing virus-1 RNA 1 (CnVYV-1 RNA 1); **(b)** CnVYV-1 RNA 2; **(c)** Cnidium vein-yellowing virus-2 RNA 1 (CnVYV-2 RNA 1); **(d)** CnVYV-2 RNA 2; **(e)** Cnidium virus X (CnVX); **(f)** Cnidium virus 1 (CnV1); **(g)** Cnidium virus 2 (CnV2). Red circles denote Korean isolates from this study; GenBank accession numbers are indicated for all taxa. Bootstrap values ≥ 50 % are shown at the nodes, and branch lengths are drawn to scale (substitutions per site).

**Supplementary Fig. S6**


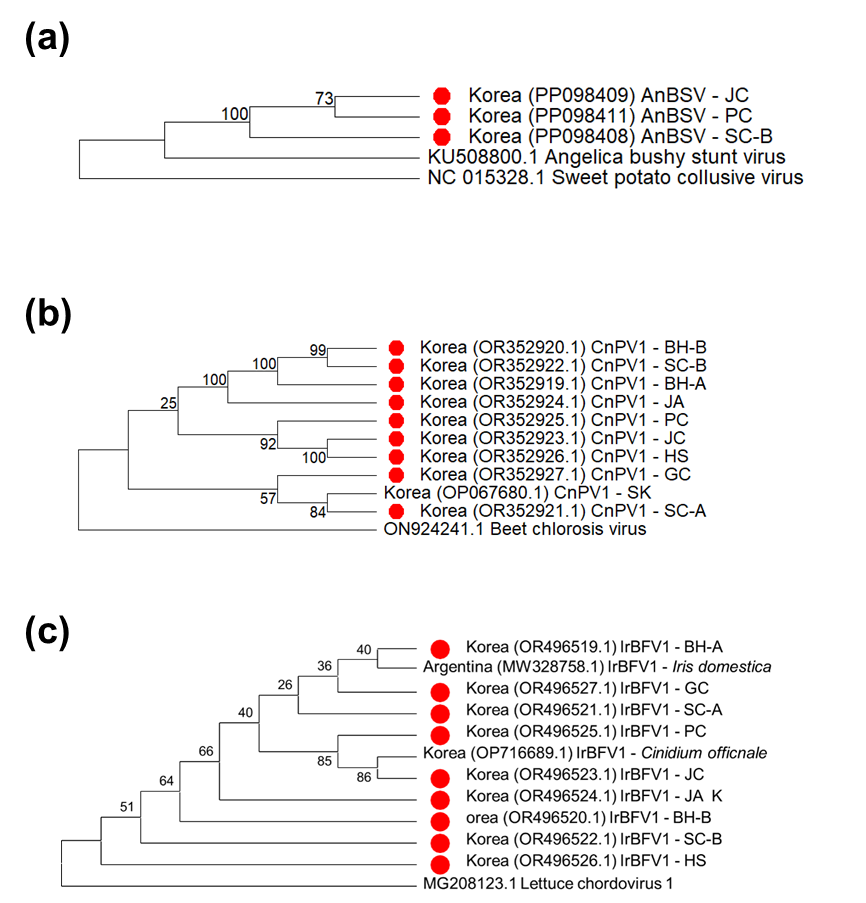

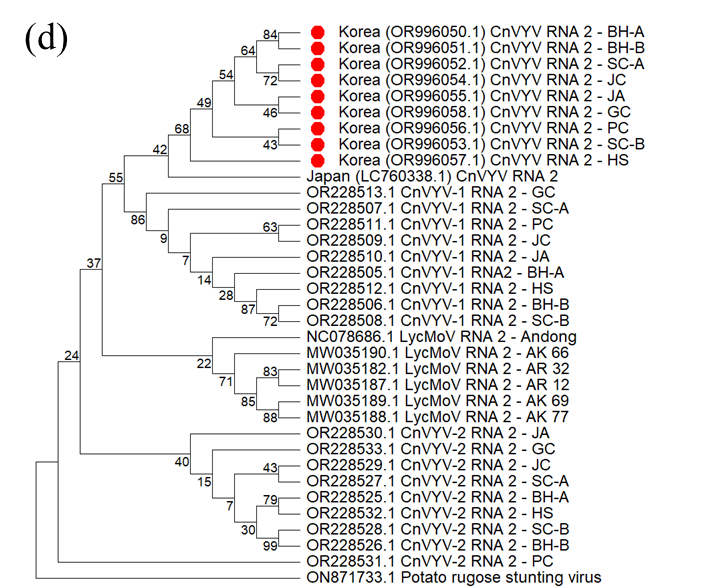


**Supplementary Fig. S6** Phylogenetic relationships of additional betaflexivirus and polerovirus isolates from *C. officinale*. Maximum-likelihood trees (1,000 bootstrap replicates) were generated from complete RdRp coding sequences. **(a)** Angelica bushy stunt virus (AnBSV); **(b)** Cnidium polerovirus 1 (CnPV1); **(c)** Iris domestica betaflexivirus 1 (IrBFV1); **(d)** Cnidium vein yellowing virus. Korean isolates are marked with red circles, and reference sequences include type strains retrieved from GenBank. Bootstrap percentages ≥ 50 % are indicated; scale bars represent nucleotide substitutions per site.

**Supplementary Table S1.** Sampling metadata and SRA accession numbers for nine *C. officinale* libraries

| Province | Locality | Isolate | BioProject (SRA Number) |
| --- | --- | --- | --- |
| Gangwon State | Hajang-myeon, Samcheok | SC-A | PRJNA1031353 |
|  | Geundeok-myeon, Samcheok | SC-B |  |
|  | Hoengseong | HS |  |
|  | Pyeongchang | PC |  |
| Gyeongsangbuk-do | Myeongho-myeon, Bonghwa | BH-A |  |
|  | Socheon-myeon, Bonghwa | BH-B |  |
| Gyeongsangnam-do | Geochang | GC |  |
| Chungcheongbuk-do | Jecheon | JC |  |
| Jeonbuk State | Jinan | JA |  |

**Supplementary Table S2.** GenBank accession numbers of all virus isolates identified in this study

|  | BH-A | BH-B | SC-A | SC-B | JC | JA | PC | HS | GC |
| --- | --- | --- | --- | --- | --- | --- | --- | --- | --- |
| CnVYV-1 RNA 1 | OR228496 | OR228497 | OR228498 | OR228499 | OR228500 | OR228501 | OR228502 | OR228503 | OR228504 |
| CnVYV-1 RNA 2 | OR228505 | OR228506 | OR228507 | OR228508 | OR228509 | OR228510 | OR228511 | OR228512 | OR228513 |
| CnVYV-2 RNA 1 | OR228516 | OR228515 | OR228517 | OR228518 | OR228519 | OR228520 | OR228521 | OR228522 | OR228523 |
| CnVYV-2 RNA 2 | OR228525 | OR228526 | OR228527 | OR228528 | OR228529 | OR228530 | OR228531 | OR228532 | OR228533 |
| CnVX | OR352928 | OR496503 | OR496504 | OR496505 | OR496506 | OR496507 | OR496508 | OR496509 | OR496510 |
| CMV RNA 1 | OR496536 | OR496537 | OR496538 | - | OR496539 | OR496540 | OR496541 | OR496542 | OR496543 |
| CMV RNA 2 | OR496544 | OR496545 | OR496546 | - | OR496547 | OR496548 | OR496549 | OR496550 | OR496551 |
| CMV RNA 3 | OR496552 | OR496553 | OR496554 | - | OR496555 | OR496556 | OR496557 | OR496558 | OR496559 |
| ASGV | OR496511 | OR496512 | OR496513 | OR496514 | OR496515 | OR496516 | OR514667 | OR496518 | OR496517 |
| CnV1 | - | OR496528 | OR496529 | OR496530 | OR496531 | OR496532 | OR496533 | OR496534 | OR496535 |
| CnV2 | OR475311 | OR475310 | OR475309 | OR475308 | OR475307 | OR475306 | OR475305 | OR475304 | OR475303 |
| CnPV1 | OR352919 | OR352920 | OR352921 | OR352922 | OR352923 | OR352924 | OR352925 | OR352926 | OR352927 |
| IrBFV1 | OR496519 | OR496520 | OR496521 | OR496522 | OR496523 | OR496524 | OR496525 | OR496526 | OR496527 |
| satCnV | PP486166 | PP486167 | PP486168 | PP486169 | PP486170 | PP486171 | PP486172 | PP486173 | PP486174 |
| CnVZ | PP076727 | PP076728 | PP076729 | PP076730 | PP076731 | PP076732 | PP076733 | PP076734 | PP076735 |
| CnVY | PP235960 | PP235961 | PP235962 | PP235963 | PP235964 | PP235965 | PP235966 | PP235967 | PP235968 |
| AnBSV | - | - | - | PP098408 | PP098409 | PP098410 | PP098411 | - | - |
| CnoV1 | PP475197 | - | PP475198 | - | - | PP475199 | PP475200 | - | PP475201 |
| CnVYV | OR996050 | OR996051 | OR996052 | OR996053 | OR996054 | OR996055 | OR996056 | OR996057 | OR996058 |

**Supplementary Table S3**. Oligonucleotide primers used for RT-PCR validation

| Virus name | Primer name | Primer sequence (5' – 3') | PCR product size |
| --- | --- | --- | --- |
| Cnidium vein yellowing virus -1 | CnVYV-1-F | GCTCTTAAGAGATCAACCAAAA | 1028 bp |
|  | CnVYV-1-R | TCCAGATTAGGCATGTTCAAG |  |
| Cnidium vein yellowing virus -2 | CnVYV-2-F | ATTTACTAGCACCGGGCTTATC | 898 bp |
|  | CnVYV-2-R | ACACCACTCGACCCACTCAT |  |
| Cnidium virus X | CnVX-F | CCATGGGATGGTTCTCACAGCAGAAGA | 718 bp |
|  | CnVX-R | CTCGAGAGGTGGGGGCAGAAGTAATA |  |
| Cucumber mosaic virus | CMV-F | CGCAGGTGGTTAACGGT | 675 bp |
|  | CMV-R | CCACACGGTAGAATCAAA |  |
| Apple stem grooving virus | ASGV-F | CTAGGCAGAACTCTTTGAACG | 261 bp |
|  | ASGV-R | CCCTTTTTGTCCTTCAGTACG |  |
| Cnidium virus 1 | CnV1-F | GCTCTTGACCCCTATGTCCG | 702 bp |
|  | CnV1-R | AACCACCCTCTCATATCCTCG |  |
| Cnidium virus 2 | CnV2-F | GTTGTGGCAAAAGAGGCAGG | 615 bp |
|  | CnV2-R | AGCTTGAGGACGAACTGTGG |  |
| Cnidium polerovirus 1 | CnPV1-F | GCGGATGCGCTGAAATTGAT | 810 bp |
|  | CnPV1-R | TCTGCTTATGGGGCTCTCCT |  |
| Iris domestica betaflexivirus 1 | IrBFV1-F | ACAAGCACCACAAGGACCAA | 707 bp |
|  | IrBFV1-R | GAGGTGGAAGCTGAGGAACC |  |
| Cnidium virus associated satellite RNA | satCnV-F | GAGACCACAAAGGGCTGTCA | 618 bp |
|  | satCnV-R | CATGAACTCGGGGATGCTGA |  |
| Cnidium virus Y | CnVY-F | CAACACCCCCGTGATCTTCA | 695 bp |
|  | CnVY-R | GCATATCCCTGTATGCCGCT |  |
| Cnidium virus Z | CnVZ-F | GTTGAAGGTGGTTGGGTTGC | 644 bp |
|  | CnVZ-R | CATTCCCCAAGGACGTGTCA |  |
| Angelica bushy stunt virus | AnBSV-F | ACCGAAACCACTAAATTCAGCTT | 730 bp |
|  | AnBSV-R | GCCAAACAAATGCTTGCACC |  |
| Cnidium officinale virus 1 | CnoV1-F | AGCTAGGATGGCATCATCTTCTT | 693 bp |
|  | CnoV1-R | ATATGGTAAGCGTTCCCATCTGA |  |
| Cnidium vein yellowing virus | CnVYV-F | TAGGGGAGGGGTTCTGGAAG | 700 bp |
|  | CnVYV-R | TAGGGGAGGGGTTCTGGAAG |  |

**Supplementary Table S4.** RT-PCR detection matrix across nine regions

|  | BH-A | BH-B | SC-A | SC-B | JC | JA | PC | HS | GC |
| --- | --- | --- | --- | --- | --- | --- | --- | --- | --- |
| CnVYV-1 | O | O | O | O | O | O | O | O | O |
| CnVYV-2 | O | O | O | O | O | O | O | O | O |
| CnVX | O | O | O | O | O | O | O | O | O |
| CMV | O | O | O | - | O | O | O | O | O |
| ASGV | O | O | O | O | O | O | O | O | O |
| CnV1 | - | O | O | O | O | O | O | O | O |
| CnV2 | O | O | O | O | O | O | O | O | O |
| CnPV1 | O | O | O | O | O | O | O | O | O |
| IrBFV1 | O | O | O | O | O | O | O | O | O |
| satCnV | O | O | O | O | O | O | O | O | O |
| CnVY | O | O | O | O | O | O | O | O | O |
| CnVZ | O | O | O | O | O | O | O | O | O |
| AnBSV | - | - | - | O | O | O | O | - | - |
| CnoV1 | O | O | O | - | - | O | O | - | O |
| CnVYV | O | O | O | O | O | O | O | O | O |

* ‘O’ = positive, ‘–’ = no amplification
